# The E3 ubiquitin-protein ligase UHRF1 promotes adipogenesis and limits fibrosis by suppressing GPNMB-mediated TGF-β signaling

**DOI:** 10.1038/s41598-024-62508-y

**Published:** 2024-05-24

**Authors:** Muneera Vakayil, Aisha Y. Madani, Maha V. Agha, Yasser Majeed, Shahina Hayat, Shameem Yonuskunju, Yasmin Ali Mohamoud, Joel Malek, Karsten Suhre, Nayef A. Mazloum

**Affiliations:** 1 College of Health and Life Sciences, Hamad Bin Khalifa University, Qatar Foundation, PO Box 34110, Doha, Qatar; 2 Department of Microbiology and Immunology, Weill Cornell Medicine-Qatar (WCM-Q), Qatar Foundation, PO Box 24144, Doha, Qatar; 3 Translational Research Institute, Academic Health System, Hamad Medical Corporation, PO Box 3050, Doha, Qatar; 4 Department of Physiology and Biophysics, Weill Cornell Medicine-Qatar (WCM-Q), Qatar Foundation, PO Box 24144, Doha, Qatar; 5 Department of Genetic Medicine, Weill Cornell Medicine-Qatar (WCM-Q), Qatar Foundation, PO Box 24144, Doha, Qatar

**Keywords:** Cell biology, Molecular biology

## Abstract

The E3 ubiquitin-ligase UHRF1 is an epigenetic regulator coordinating DNA methylation and histone modifications. However, little is known about how it regulates adipogenesis or metabolism. In this study, we discovered that UHRF1 is a key regulatory factor for adipogenesis, and we identified the altered molecular pathways that UHRF1 targets. Using CRISPR/Cas9-based knockout strategies, we discovered the whole transcriptomic changes upon UHRF1 deletion. Bioinformatics analyses revealed that key adipogenesis regulators such PPAR-γ and C/EBP-α were suppressed, whereas TGF-β signaling and fibrosis markers were upregulated in UHRF1-depleted differentiating adipocytes. Furthermore, UHRF1-depleted cells showed upregulated expression and secretion of TGF-β1, as well as the glycoprotein GPNMB. Treating differentiating preadipocytes with recombinant GPNMB led to an increase in TGF-β protein and secretion levels, which was accompanied by an increase in secretion of fibrosis markers such as MMP13 and a reduction in adipogenic conversion potential. Conversely, UHRF1 overexpression studies in human cells demonstrated downregulated levels of GPNMB and TGF-β, and enhanced adipogenic potential. In conclusion, our data show that UHRF1 positively regulates 3T3-L1 adipogenesis and limits fibrosis by suppressing GPNMB and TGF-β signaling cascade, highlighting the potential relevance of UHRF1 and its targets to the clinical management of obesity and linked metabolic disorders.

## Introduction

The prevalence of obesity continues to rise at an alarming rate; about 40% of the global population is overweight, and 13% are obese^1^. Excessive fat accumulation increases the risk of cardiovascular diseases, diabetes, musculoskeletal disorders, and certain cancers^2^; therefore, it is essential to understand the mechanism of fat mass expansion observed in obese states to develop better therapeutic strategies. During nutrient excess, adipose tissue stores excess energy by expanding through the enlargement of existing adipocytes (hypertrophy) and/or by forming new adipocytes from precursors (hyperplasia), the latter being associated with less inflammation and improved insulin sensitivity^3^. Besides storing lipids, the adipose tissue acts as an endocrine organ that secretes adipokines such as leptin and adiponectin, which regulate diverse processes such as energy expenditure, appetite, and glucose metabolism^4^.

Adipogenesis, the conversion of preadipocytes to adipocytes, has been studied extensively because of its significance to energy metabolism^5^, and a widely used in vitro model system is 3T3-L1 preadipocytes^6^. Upon stimulation with a differentiation cocktail containing insulin, 1-methyl-3-isobutyl xanthine (IBMX), and dexamethasone, growth-arrested 3T3-L1 preadipocytes undergo synchronous re-entry into the cell cycle and mitotic clonal expansion (MCE) followed by mitotic exit and terminal differentiation to mature adipocytes^6–8^. Different transcription factors such as CCAAT/enhancer-binding protein-β (C/EBP-β), C/EBP-δ, Peroxisome Proliferator-Activated Receptor gamma (PPAR-γ), and C/EBP-α are involved in adipogenesis^9,10^, and these factors upregulate key enzymes that are required for triglyceride accumulation, such as fatty acid synthase, fatty acid binding protein 4 (FABP4), sterol regulatory element-binding protein 1 (SREBP-1) and lipoprotein lipase (LPL)^11^. Epigenetic regulators may also control adipogenesis by modifying the transcriptional landscape of adipocyte-specific genes through mechanisms like DNA methylation and post-translational histone modifications^12^. UHRF1 (Ubiquitin-like containing PHD Ring Finger, inverted CCAAT box-binding protein of 90 kDa [ICBP90] or Np95) is a hub protein involved in integrating epigenetic information, cell cycle regulation, and proliferation^13^, but we know little about its function in adipogenesis. UHRF1-interacting proteins such as Tip60^14^, USP7^15^, HDAC1, and DNMT1^16^ have been reported to regulate adipogenesis^12,17,18^. For instance, the expression of Tip60, a member of the MYST family of acetyltransferases, and USP7, a deubiquitinase, increases during the initial stages of 3T3-L1 differentiation. USP7 increases the stability of Tip60 by deubiquitylation and regulates the expression of early phase adipogenesis genes, including C/EBPs^17^. Similarly, histone deacetylase (HDAC) inhibitors can efficiently block in vitro adipocyte differentiation and enhance osteogenesis in mesenchymal stem cell (MSCs)^18^. These reports highlight that UHRF1 may regulate adipogenesis. The potential role of UHRF1 in cytokine secretion through epigenetic alterations leading to inflammation and metabolic disorders has also been reported. UHRF1-deficient macrophages in zebrafish overexpress TNF-α^19^. Increased TNF-α expression was observed in UHRF1-deficient transgenic mice, demonstrating the suppression of TNF-α by DNMT1-dependent DNA methylation on its genome mediated by UHRF1^20^. Additionally, UHRF1 deletion in mature T-regulatory cells also leads to inflammation in multiple organs^21^.

Adipose tissue expansion also relies on extracellular matrix (ECM) protein remodeling^22^. ECM comprises many structural and adhesive proteins, such as collagens, fibronectin, laminins, and proteoglycans. However, excess collagen and fibronectin deposits can restrict adipocyte expansion, which leads to adipocyte necrosis and pro-inflammatory macrophage infiltration^23^. A hallmark of obesity-related metabolic disorders is the secretion of inflammatory cytokines and other factors from adipocytes^4,24,25^. For instance, elevated TGF-β level in obese subjects^26^ is associated with a poor metabolic profile, hypertension, T2D, fibrosis, inflammation, and ECM remodeling^27–29^. Diet-induced obesity (DIO) and insulin resistance are associated with increased circulating levels of GPNMB^30^. GPNMB (osteoactivin/DC-HIL/HGFIN) is a type I transmembrane glycoprotein present in different cell types, including bone, immune cells, skin, and hematopoietic cells^31,32^. The extracellular domain (ECD) of GPNMB comprises glycosylation sites, signal peptide domain (SIG), integrin-binding RGD motif (NTD), polycystic kidney disease domain, and Kringle-like domain (KLD)^33^. The cytoplasmic domain of GPNMB has an immunoreceptor tyrosine-based activation motif (hemITAM) and a di-leucine motif for intracellular signaling and lysosomal/endosomal targeting^34^. Secreted GPNMB can bind to different receptors and mediate multi-faceted functions such as bone remodeling, tissue regeneration, modulation of inflammation, and cancer inhibition/metastasis^31,33^. Recent studies have shown that GPNMB is associated with systemic metabolic dysfunction and lysosomal storage disorders^35^. When GPNMB signaling was inhibited with a neutralizing antibody, insulin sensitivity in obese mice improved^30^. These findings provide a rationale for focusing on peptide-based therapeutics for adipocyte/lipid metabolism dysfunction.

In this study, we found that UHRF1 is essential for adipogenesis and is a negative regulator of fibrosis and inflammation in 3T3-L1 (pre)adipocytes. UHRF1-depletion led to impaired lipid accumulation, reduced expression of adipocyte markers like PPAR-γ, C/EBP-α, and GLUT4, and increased expression of GPNMB and TGF-β. Conversely, UHRF1 overexpression downregulated GPNMB and TGF-β levels in HEK293T cells and promoted adipogenesis in SW872 human preadipocytes. These results suggest that UHRF1 has a regulatory role in adipogenesis, fibrosis, and inflammation, which may have therapeutic value in treating obesity and its related comorbidities.

## Results

### UHRF1-depletion suppresses adipogenesis differentiation in 3T3-L1 preadipocytes

To investigate the role of UHRF1 in adipogenesis, we deleted or silenced the UHRF1 gene in 3T3-L1 cells and induced them with a chemical cocktail to stimulate adipogenic differentiation, and the comparison between the effect of the function loss of UHRF1 in 3T3-L1 to control cells was studied. We used 2 UHRF1 targeting guide RNAs (gRNAs) and 1 scrambled gRNA in CRISPR/Cas9 technology to generate UHRF1 knockout gRNA 1 (UHRF1 KO G1), UHRF1 knockout gRNA 2 (UHRF1 KO G2) and Non-Targeting (NT) cells. Next, we used 2 shRNA constructs targeting UHRF1 mRNA and 1 scrambled construct in the lentiviral shRNA method to generate UHRF1 knockdown construct 1 (shUHRF1 C1), UHRF1 knockdown construct 2 (shUHRF1 C2) cells, and shScramble. NT and shScramble did not encode any sequence complementarity to the mouse genome, so we used them as controls.

**Figure 1:**
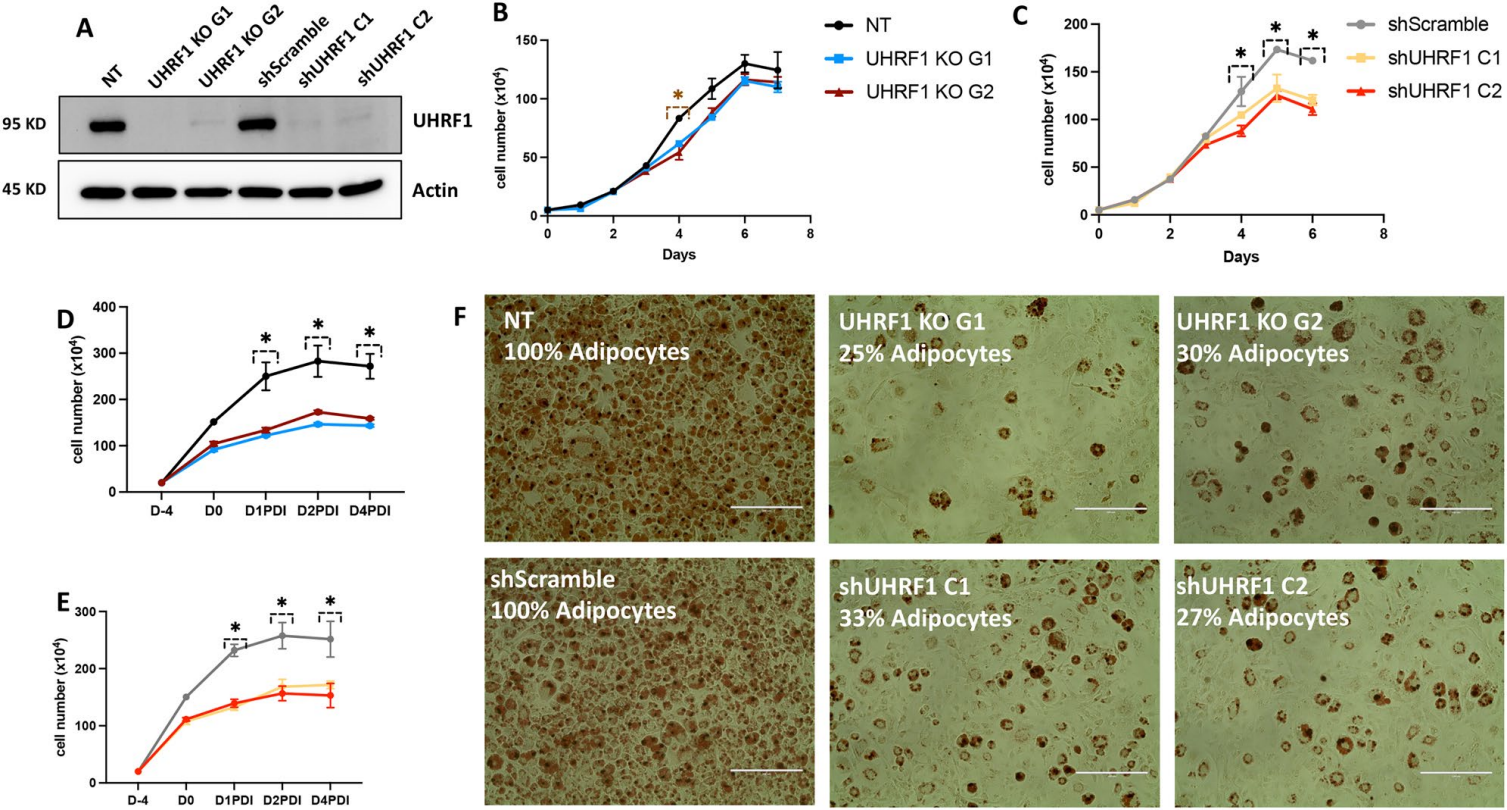
UHRF1 is required for 3T3-L1 adipogenesis. (**A**) Western blot analysis of UHRF1 protein expression in NT, UHRF1 knockout G1 (UHRF1 KO G1), UHRF1 knockout G2 (UHRF1 KO G2), shScramble, shUHRF1 C1 and shUHRF1 C2 3T3-L1 cells, with Actin as the loading control. (**B**, **C**) Graph representing the growth curve of NT, UHRF1 KO G1, UHRF1 KO G2, shScramble, shUHRF1 C1, and shUHRF1 C2 preadipocytes at different time points. (**D**, **E**) Cell count of NT, UHRF1 KO G1, UHRF1 KO G2, shScramble, shUHRF1 C1, and shUHRF1 C2 cells during adipogenic differentiation. (**F**) Oil Red O staining of adipocytes at day 10 post-differentiation in NT, UHRF1 KO G1, UHRF1 KO G2, shScramble, shUHRF1 C1, and shUHRF1 C2 cells visualized by light microscopy at ×20 magnification (scale bar, 200 μm). The percentage of adipocytes in each group is indicated in the image. Data are mean ± SEM of 3 independent experiments. Statistical analyses were performed using ordinary one-way ANOVA, *P < 0.05.

We validated successful knockout and knockdown by using Western blotting to analyze UHRF1 protein levels in total lysates prepared from the control and UHRF1-depleted preadipocytes with Actin probed as the loading control. The results showed undetectable levels of UHRF1 protein in UHRF1 KO G1 and UHRF1 KO G2 compared to NT and in shUHRF1 C1 and shUHRF1 C2 compared to shScramble (Fig. 1A). When we analyzed the growth curve of NT, UHRF1 KO G1, UHRF1 KO G2, shScramble, shUHRF1 C1, and shUHRF1 C2, we found that the growth rate of UHRF1-depleted cells decreased compared to their respective controls (Fig. 1B, C). Next, to investigate the consequence of UHRF1-depletion in adipogenesis, we stimulated the above groups of cells with standard adipogenic media and monitored differentiating cells for cell counts. As illustrated in Fig. 1D, day 2 cell counts of UHRF1 KO G1 and UHRF1 KO G2 showed 58% and 48% fewer cells than NT. Likewise, shUHRF1 C1 and shUHRF1 C2 showed a decrease in cell number of 34% and 35% respectively, compared to the cell counts of shScramble on day 2, indicating impaired MCE in UHRF1-depleted cells (Fig. 1E). UHRF1-depleted cells exhibited decreased terminal differentiation on day 10 post-induction, as evidenced by reduced lipid accumulation after Oil Red O staining (Fig. 1F). We counted lipid-laden cells manually to quantify Oil Red O images. In UHRF1 KO G1 and UHRF1 KO G2 cells, the differentiation percentage was 25% and 30%, respectively, in contrast to 100% differentiation in NT. Likewise, shUHRF1 C1 and shUHRF1 C2 cells showed only 33% and 27% differentiation respectively, compared to 100% in shScramble (Fig. 1F). These results demonstrate that adipogenesis required UHRF1, and UHRF1-deficiency led to impaired lipid accumulation in 3T3-L1 cells. Technically, UHRF1-depletion in 3T3-L1 cells was more efficient with UHRF1 KO G1 and shUHRF1 C2 constructs compared to others, so we selected these constructs for subsequent experiments.

### Transcriptomics analysis of UHRF1 knockout versus non-targeted (pre)adipocytes identifies key differentially expressed genes and altered pathways, including those linked to adipocyte differentiation and fibrosis

**Figure 2:**
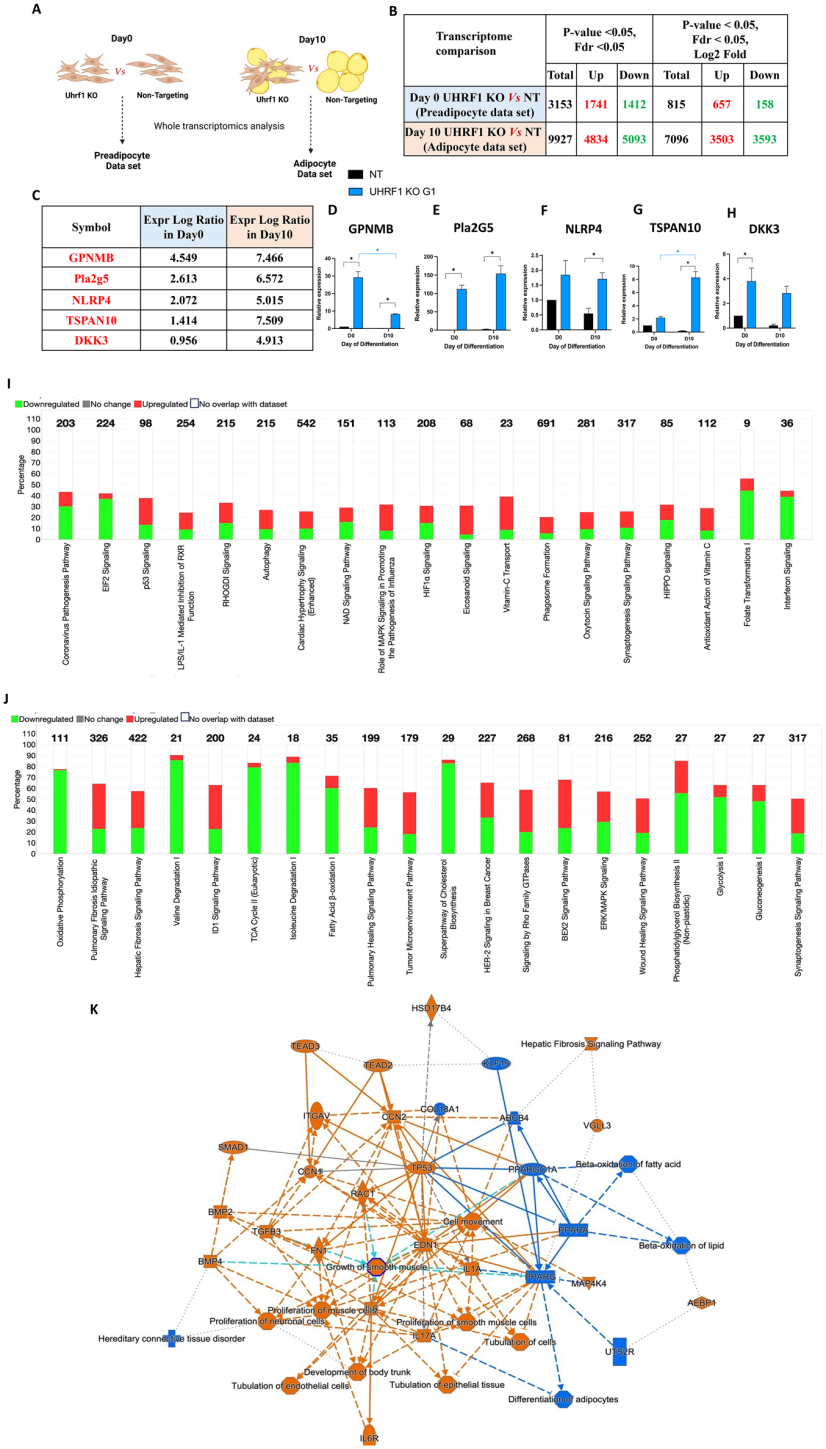
Transcriptomics analysis of UHRF1 KO G1 (pre)adipocytes reveals DEGs and significantly altered pathways, including inhibition of adipocyte differentiation. (**A**) Schematic representation of transcriptomics analysis of whole RNA extracted from the following samples: UHRF1 KO G1 versus NT at preadipocyte stage (day 0) and UHRF1 KO versus NT at adipocyte stage (day 10) of 3T3-L1 cells. (**B**) The number of statistically significant DEGs in both datasets at the indicated cut-off *P*-value < 0.05 and FDR < 0.05. Upregulated and downregulated genes are highlighted in red and green, respectively. (**C**–**H**) Expression log ratio of top upregulated genes in both data sets of UHRF1 KO cells and its qPCR validation. (**I**, **J**) IPA prediction of the top 20 affected canonical pathways in both datasets, and those highlighted in red and green are the percentage of upregulated and downregulated genes in each pathway, respectively. (**K**) Summary graph of adipocyte dataset that shows the interaction network between the most significant predicted pathways and molecules. The orange line indicates activated entities with a positive z-score, and the blue line indicates inhibited entities with a negative z-score, respectively, and estimated edges are shown as dotted lines. Data are mean ± SEM of 3 independent experiments. Statistical analyses were performed using ordinary one-way ANOVA, *P < 0.05.

To discover molecular and transcriptional pathways regulated by UHRF1 in adipogenesis, we performed comparative transcriptomics analysis (RNA-seq) on total RNA collected from UHRF1 KO and NT (pre)adipocytes. We collected RNA from cells on day 0 (non-induced) and day 10 post-induction, representing the preadipocyte and adipocyte datasets respectively (Fig. 2A). Principal component analysis (PCA) of the transcriptomics data revealed distinct sample clusters representing a unique gene expression pattern in each of the 4 experimental groups (Supplementary Fig. 1). Next, we identified differentially-expressed genes (DEGs) with a false discovery rate (FDR) of < 0.05 and a *P*-value cut-off of 0.05. We identified 3153 DEGs in the preadipocyte dataset with 1741 upregulated genes and 1412 downregulated genes. We identified 9927 DEGs in the UHRF1 KO adipocyte dataset with 4834 upregulated genes and 5093 downregulated genes. For Ingenuity Pathway Analysis (IPA) enrichment analysis of the day 10 adipocyte dataset, we filtered the genes to 7096 genes with Log_2_ fold change (Fig. 2B). Next, we validated some of the top DEGs in both datasets by qPCR, including genes such as TSPAN10, NLRP4, Pla2G5, DKK3, and GPNMB (Fig. 2C–H). We identified the most affected canonical pathways in UHRF1-lacking cells using QIAGEN IPA based on a cut-off *P*-value of 0.05. The most significantly downregulated pathways in the preadipocyte dataset included EIF2 signaling, NAD^+^ signaling, folate transformations, and interferon signaling. The most significantly upregulated pathways in the preadipocyte dataset included p53, LPS-mediated inhibition of RXR, autophagy, cardiac hypertrophy, MAPK signaling, eicosanoid signaling, and phagosome formation (Fig. 2I). The most significantly downregulated canonical pathways in the adipocyte dataset included oxidative phosphorylation, amino acid (valine, isoleucine) degradation pathways, TCA cycle, fatty acid beta-oxidation, super-pathway of cholesterol biosynthesis, glycolysis, gluconeogenesis, and phosphatidyl glycerol biosynthesis. Interestingly, most of these pathways are closely connected to adipogenesis, so their downregulation in UHRF1-null adipocytes confirms their limited adipogenic potential. The upregulated pathways in UHRF1-null adipocytes included those linked with pulmonary fibrosis, hepatic fibrosis, ID1 signaling, pulmonary healing pathway, signaling by Rho Family GTPase, and Bex2 signaling (Fig. 2J).

Next, we performed a graphical summary analysis for insights into how the top significant entities and predicted biological pathways interact. In the preadipocyte dataset, the significantly activated networks in the summary graph were mainly cell movement of macrophage, antigen presenting cells, phagocytes, and tumor cells, increased microtubule dynamics, formation of cellular protrusions, hepatic and pulmonary fibrosis, cell interaction, and cell invasion. In these networks, upregulated molecules included ACKR2, TRIM24, PNPT1, AGT, IGF1, FGF2, PIK3CG, AGT, EPAS1, and OLR1, and downregulated molecules included IRF7, IRF3, DDX58, MAVS, STAT1, IFNA2, IFNL1, and PARP9 (Supplementary Fig. 2). In the adipocyte dataset, the significantly activated networks were mainly hepatic fibrosis, cell movement, tubulation of epithelial and endothelial tissue, growth and proliferation of smooth muscle and neuronal cells, and their associated molecules, including TGFB3, BMP2, BMP4, SMAD1, FN1, TP53, CCN1, CCN2, ITGAV, IL6R, RAC1, etc. The pathways that were predicted to be significantly inhibited in the adipocyte dataset include differentiation of adipocytes, beta-oxidation of lipid and fatty acids, mitochondrial dysfunction, and connective tissue disorder, in which the correlated downregulated genes include PPAR-γ, PPARGC1α, PPAR-α, KLF15, ABCB4 and COL18A1 (Fig. 2K). In conclusion, we uncovered key DEGs and molecular pathways altered in UHRF1-null cells using transcriptomics analysis, which offered insights into candidate adipogenesis regulators with therapeutic potential for metabolic diseases.

### UHRF1 expression is induced early during differentiation and positively regulates adipogenesis

**Figure 3:**
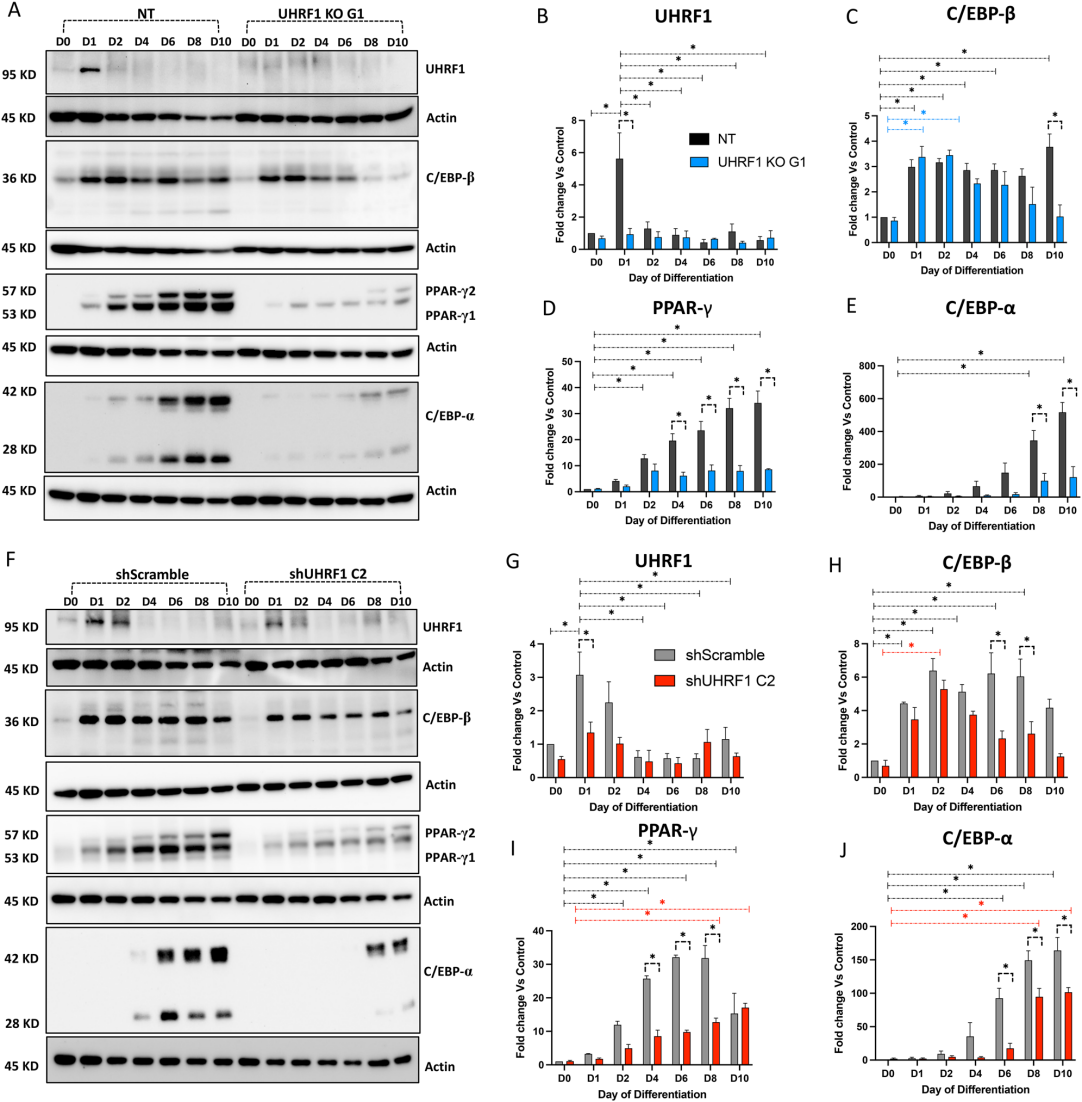
Adipogenic differentiation is suppressed in UHRF1-depleted cells. (**A**) Western blot analysis of UHRF1 and adipocyte markers C/EBP-β, PPAR-γ, and C/EBP-α in NT and UHRF1 KO G1 at different time points during adipogenic differentiation. (**B**–**E**) Western blot quantification of UHRF1, C/EBP-β, PPAR-γ, and C/EBP-α in NT and Uhrf1 KO G1 in which relative protein level was normalized to the loading control, Actin, using ImageJ software. (**F**–**J**) Western blot analysis of UHRF1 and adipogenic markers C/EBP-β, PPAR-γ, and C/EBP-α and its quantification graph in shScramble and shUHRF1 C2 during adipogenic differentiation. Data are mean ± SEM of 3 independent experiments. Statistical analyses were performed using ordinary one-way ANOVA, *P < 0.05.

To evaluate the significance of UHRF1 in adipogenesis, we examined its expression pattern during adipocyte differentiation. In NT and shScramble cells, UHRF1 expression showed a significantly rapid induction on day 1, followed by a return to basal levels during later stages of differentiation. As expected, shUHRF1 C2 cells showed lower basal levels in UHRF1 mRNA expression than shScramble (Supplementary Fig. 3A). By western blot analysis, we observed a 5.6-fold and 3-fold increase in the expression of UHRF1 in NT (Fig. 3A, B) and shScramble (Fig. 3F–G) respectively on day 1. However, UHRF1 KO G1 and shUHRF1 C2 demonstrated only a 0.93-fold and 1.34-fold increase in UHRF1 expression at the same time. These results suggest that UHRF1 is upregulated in the MCE phase of differentiation.

Adipogenesis is a multistep process coordinated by a specific set of molecules, including transcription factors. Among them, the elevation and activation of C/EBP-β, which occurs in the early phase of differentiation, is followed by the sequential activation of various adipogenesis markers, such as PPAR-γ, the master regulator of adipogenesis, and C/EBP-α. Therefore, to validate the inhibition of adipogenesis in UHRF1 KO transcriptomics dataset, we examined the expression of these adipocyte-linked markers by qPCR and western blot methods using samples collected at different times during differentiation from control cells and UHRF1-depleted cells. UHRF1 deletion did not significantly affect the early induction of C/EBP-β in UHRF1 KO G1 compared to NT during differentiation. C/EBP-β mRNA was also induced at similar levels in both NT and UHRF1 KO G1 (Supplementary Fig. 4B). However, UHRF1 KO G1 adipocytes showed lower levels of C/EBP-β protein during the late differentiation phase, with a significant difference observed on day 10 (Fig. 3C). We obtained similar results using the shScramble and shUHRF1 C2 samples in which, although C/EBP-β mRNA levels were similarly induced in shScramble and shUHRF1 C2 (Supplementary Fig. 3B), there were significantly lower levels of C/EBP-β protein in shUHRF1 C2 during the late phase of differentiation (Fig. 3H).

Levels of other adipogenesis markers, PPAR-γ and C/EBP-α, were also significantly lower in UHRF1-depleted cells compared to their respective controls. Specifically, PPAR-γ mRNA expression was 49.53% (Supplementary Fig. 4C), and the PPAR-γ protein expression was 24.69% (Fig. 3D) in UHRF1 KO G1 compared to NT PPAR-γ expression on day 8. Similarly, in shUHRF1 C2, PPAR-γ mRNA expression was 67.67% (Supplementary Fig. 3C), and PPAR-γ protein expression was 40% (Fig. 3I) compared to the PPAR-γ expression in shScramble on day 8. Likewise, C/EBP-α expression was 22.59% at the mRNA level (Supplementary Fig. 4D) and 28.8% at the protein level (Fig. 3E) in UHRF1 KO G1 compared to the expression in NT on day 8. C/EBP-α expression in shUHRF1 C2 was 83.04% at mRNA level (Supplementary Fig. 3D) and 63.64% at protein level (Fig. 3J) compared to its expression in shScramble on day 8. Together, these results suggest that UHRF1 is a positive regulator of adipogenesis, and in its absence, adipocyte markers are suppressed, and adipogenesis is impaired.

### UHRF1-depletion is associated with GPNMB induction and secretion

**Figure 4:**
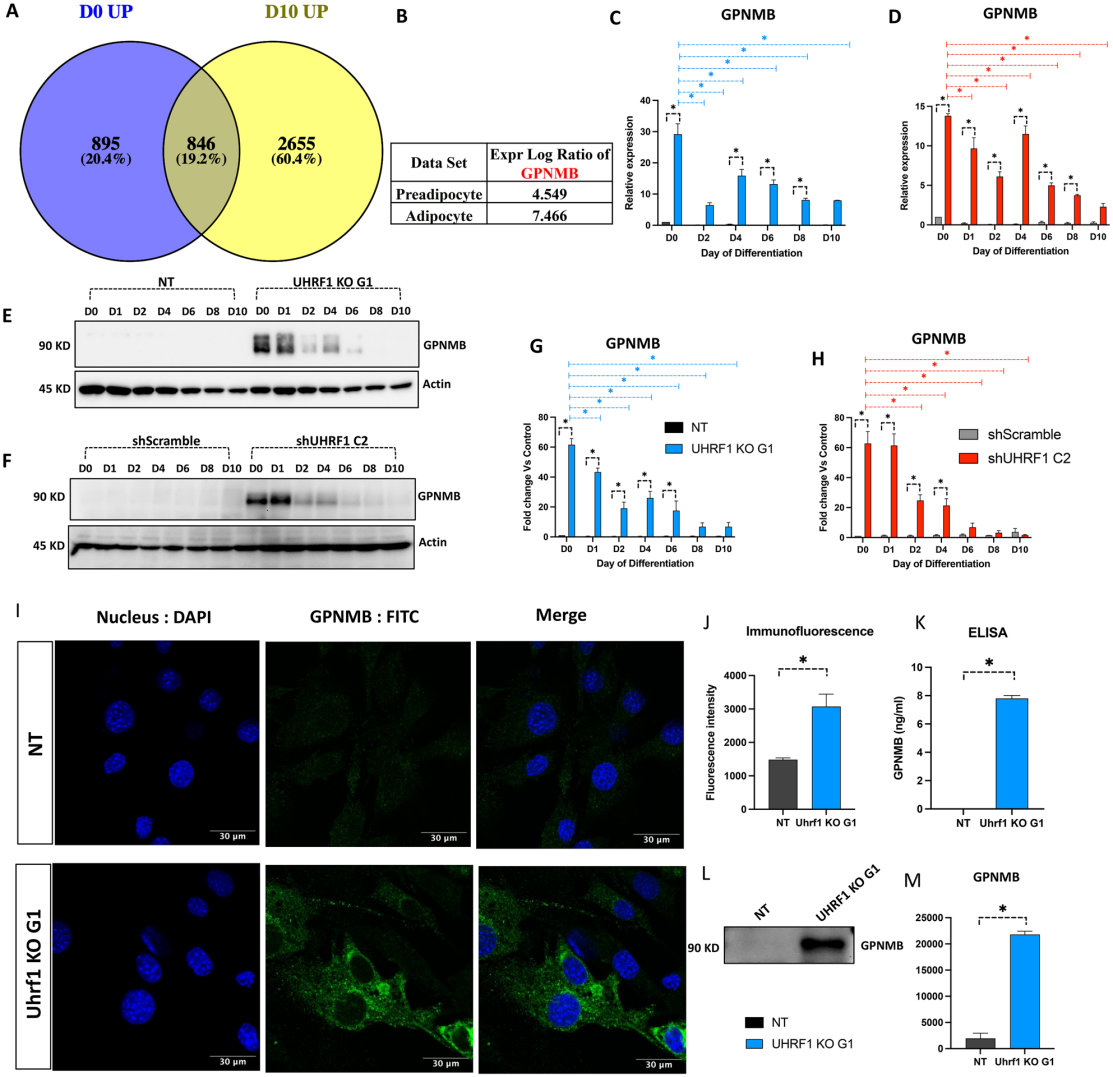
GPNMB is induced and secreted in UHRF1-lacking 3T3-L1 preadipocytes. (**A**) Venn diagram showing 846 common upregulated genes between preadipocyte and adipocyte data sets. (**B**) The Expression log ratio of GPNMB in both datasets. (**C**, **D**) Relative mRNA expression of GPNMB in NT, UHRF1 KO G1, shScramble, and shUHRF1 C2 at different time-points during adipocyte differentiation by qPCR. (**E**, **F**) GPNMB protein levels in NT, UHRF1 KO G1, shScramble, and shUHRF1 C2 by Western blot analysis with Actin as the loading control. (**G**, **H**) ImageJ software-based Quantitation of GPNMB protein in Western blot results by normalizing to Actin. Fold change values were calculated relative to the D0 control. (**I**) Immunostaining of GPNMB in NT, UHRF1 KO G1 preadipocytes where nuclei are stained in blue (DAPI), GPNMB stained in green (FITC). Images were taken at 63 × magnification. (**J**) Quantification of GPNMB fluorescence intensity in NT and UHRF1 KO G1 preadipocytes immunostaining by ImageJ software. (**K**) ELISA detected the secreted level of GPNMB in the conditioned media of NT and UHRF1 KO G1 preadipocytes. (**L**, **M**) Western blot analysis of GPNMB and its quantification graph in the conditioned media of NT and UHRF1 KO G1 preadipocytes. Data are mean ± SEM of 3 independent experiments. Statistical analyses were performed using ordinary one-way ANOVA, *P < 0.05.

To identify the common DEGs and altered pathways between UHRF1 KO preadipocytes and adipocytes, we compared both datasets by Venn diagram analysis. Of the 1412 downregulated DEGs in UHRF1 KO preadipocytes and 3593 downregulated DEGs in UHRF1 KO adipocytes, 281 genes were common (Supplementary Fig. 5A). Similarly, among the 1741 upregulated genes in UHRF1 KO preadipocytes and 3503 upregulated genes in UHRF1 KO adipocytes, 846 genes were common (Fig. 4A). We then subjected the common genes to the Kyoto Encyclopedia of Genes and Genomes (KEGG) pathway analysis to enrich the pathways associated with DEGs at *P*-value cut-off 0.05. The significant pathways associated with common downregulated genes included carbon metabolism, metabolic pathways, amino acid biosynthesis, fatty acid metabolism, HIF-1 signaling, amino acid degradation, and glycolysis (Supplementary Fig. 5B). The significant pathways associated with common upregulated genes included lysosome, glycosamino glycan degradation, ECM-receptor interaction, focal adhesion, phagosome, and proteoglycans in cancer (Supplementary Fig. 5C). Next, we identified the top affected molecules by analyzing the log_2_ ratio of shared genes separately in both the preadipocyte and adipocyte datasets. This analysis led us to identify prolactin family 2 (Prl2c2) as one of the most downregulated genes in both datasets (Supplementary Fig. 5A) and glycoprotein non-metastatic gene B (GPNMB) as the most upregulated gene in both datasets (Fig. 4B). Interestingly, most of the top enriched pathways in KEGG analysis, including lysosome^35–37^, proteoglycans in cancer^38^, focal adhesion^39^, ECM-receptor interaction^39^ and PI3K-AKT signaling^40^ had been reported for their correlation with GPNMB signaling.

GPNMB is a transmembrane glycoprotein involved in various processes, including immune modulation^41,42^. However, the notion that GPNMB enhances the pathogenicity of obesity^41,43^ or limits obesity-associated inflammation^42^ is controversial. Therefore, we investigated the functional role of GPNMB in UHRF1 KO cells. Consistent with the transcriptomics results, GPNMB mRNA was significantly induced in UHRF1 KO G1 (Fig. 4C) and shUHRF1 C2 (Fig. 4D) compared to their respective controls, NT and shScramble. Western blot analysis showed significant GPNMB induction in UHRF1 KO G1 (Fig. 4E, G) and shUHRF1 C2 (Fig. 4F, H). Specifically, GPNMB-induction was higher during the initial days of differentiation, followed by a decline. However, UHRF1-depleted cells maintained higher GPNMB expression levels than controls throughout. Using confocal microscopy, we found that GPNMB was expressed at higher levels in the cytoplasm of UHRF1 KO cells compared to NT (Fig. 4I, J). To test if these cells secreted GPNMB, after normalization to total protein concentration, we assessed the level of GPNMB in the conditioned medium collected from UHRF1 KO G1 preadipocytes and NT cells (Supplementary Fig. 6A). As measured by ELISA, the level of soluble GPNMB in the conditioned media of UHRF1 KO G1 showed ~ 8 ng/ml GPNMB, whereas it was undetectable in the conditioned media of NT (Fig. 4K). Western blot analysis of conditioned media also revealed GPNMB in UHRF1 KO G1 cells, showing enhanced secretion by UHRF1 KO G1 preadipocytes compared to the control (Fig. 4L, M). The soluble form of GPNMB can be secreted by various mechanisms, including through cleavage by a disintegrin and metallopeptidase domain-containing protein 10 (ADAM10)^44^. Though the levels of ADAM10 mRNA were induced during differentiation, there was no significant difference in the expression levels of ADAM10 in UHRF1 KO G1 compared to NT (Supplementary Fig. 6B). Therefore, further experiments are needed to investigate the mechanism of GPNMB secretion. In summary, the above experiments validated the robust induction of GPNMB at the transcript and protein levels in UHRF1-null 3T3-L1 cells.

### TGF-β and its targets are induced in UHRF1 KO cells during differentiation

**Figure 5:**
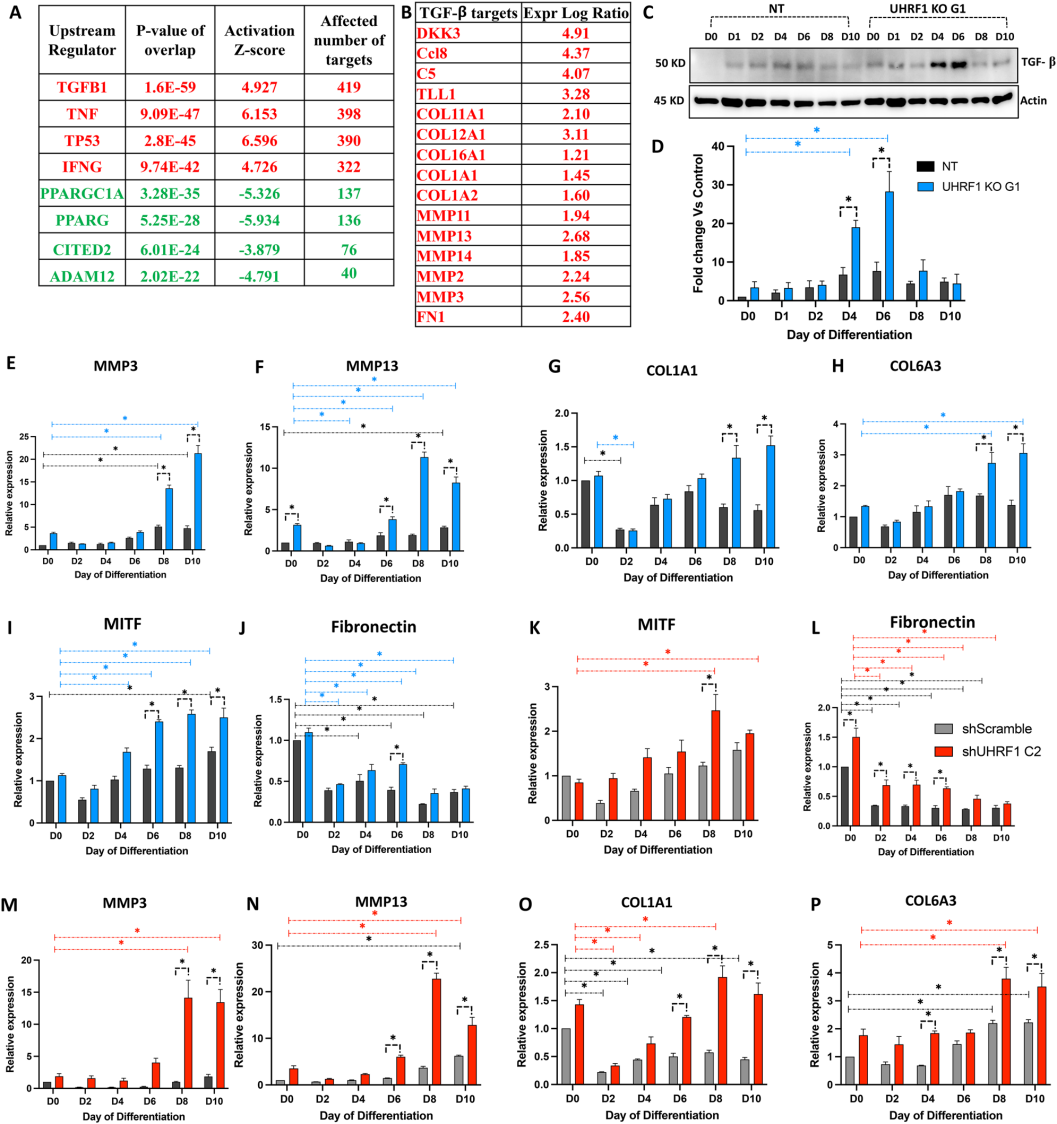
TGF-β and its targets are induced in UHRF1 KO cells during differentiation. (**A**) Upstream regulators of common upregulated genes predicted by IPA analysis. Upregulated targets are represented in red color, and downregulated targets are shown in green color. (**B**) Upregulated TGF-β targets predicted in adipocyte dataset. (**C**, **D**) Western blot analysis showing TGF-β induction in NT and UHRF1 KO G1 during differentiation and its quantification graph. (**E**–**P**) qPCR analysis of MMP3, MMP13, COL1A1, COL6A3, MCP1, Fibronectin, and MITF in NT, UHRF1 KO G1 and shUHRF1 C2 cells during differentiation. Data are mean ± SEM of 3 independent experiments. Statistical analyses were performed using ordinary one-way ANOVA, *P < 0.05.

We subjected the common upregulated genes from the transcriptomics dataset to IPA enrichment analysis to gain mechanistic insights into the molecular pathways potentially regulated by UHRF1 during adipogenesis. We identified potential upstream regulators by applying a *P*-value of overlap < 0.05 threshold. We enriched 209 upstream regulators with Z-scores ≥ 2.5, and among them, the most significantly activated upstream regulator was TGF-β 1 (Z-score = 4.927; *P*-value of overlap = 1.6E−59), followed by TNF, TP53, and IFN-γ. The most inhibited upstream regulator with Z-scores ≤ − 2.5 was PPARGCI-α (Z-score = − 5.326; *P*-value of overlap = 3.28E−35), followed by PPAR-γ, CITED2, ADAM12 (Fig. 5A). Many studies have reported TGF-β mediated adipogenesis inhibition^29^. Therefore, we evaluated the common TGF-β targets in UHRF1 KO preadipocyte and adipocyte datasets to investigate the mechanisms of adipogenic suppression. Our analysis identified 328 common TGF-β targets, which included CEBPα, ADIPOQ, CIDEC, TIMP3, MMP2, MMP3, MMP13, MMP14, CD36, and FABP5, among others. Next, to functionally validate TGF-β activation, we performed western blotting using NT and UHRF1 KO G1 protein lysates collected at different times during differentiation. We found a significant induction of TGF-β on days 4 and 6 of differentiation in UHRF1 G1 KO compared to NT (Fig. 5C, D). Upregulated targets were mainly correlated to fibrosis and ECM genes including COL11A1, COL12A1, COL16A1, COL1A1, COL1A2, MMP2, MMP3, MMP11, MMP13, MMP14 and FN1 (Fig. 5B). Downregulated TGF-β targets in the UHRF1 KO adipocyte dataset included adipocytes markers such as PPAR-γ, CEBP-α, ADIPOQ, Resistin, VEGF, FABP5, and CD36, suggesting the molecular suppression of adipogenesis in them. Next, we validated the expression of some common TGF-β targets by qPCR analysis and found significantly increased MMP3, MMP13, COL1A1, COL6A3, fibronectin, and MITF in both UHRF1 KO G1 and shUHRF1 C2 during differentiation compared to the controls, NT and shScramble (Fig. 5E–P). Most targets were significantly induced during the late differentiation phase, suggesting that the upregulation was linked to TGF-β activation in the UHRF1-depleted cells. In summary, these results show that TGF-β and its targets are upregulated in the absence of UHRF1, which might lead to inhibitory effects on adipogenesis.

### Recombinant GPNMB treatment induces TGF-β and suppresses adipogenic differentiation in 3T3-L1 cells

**Figure 6:**
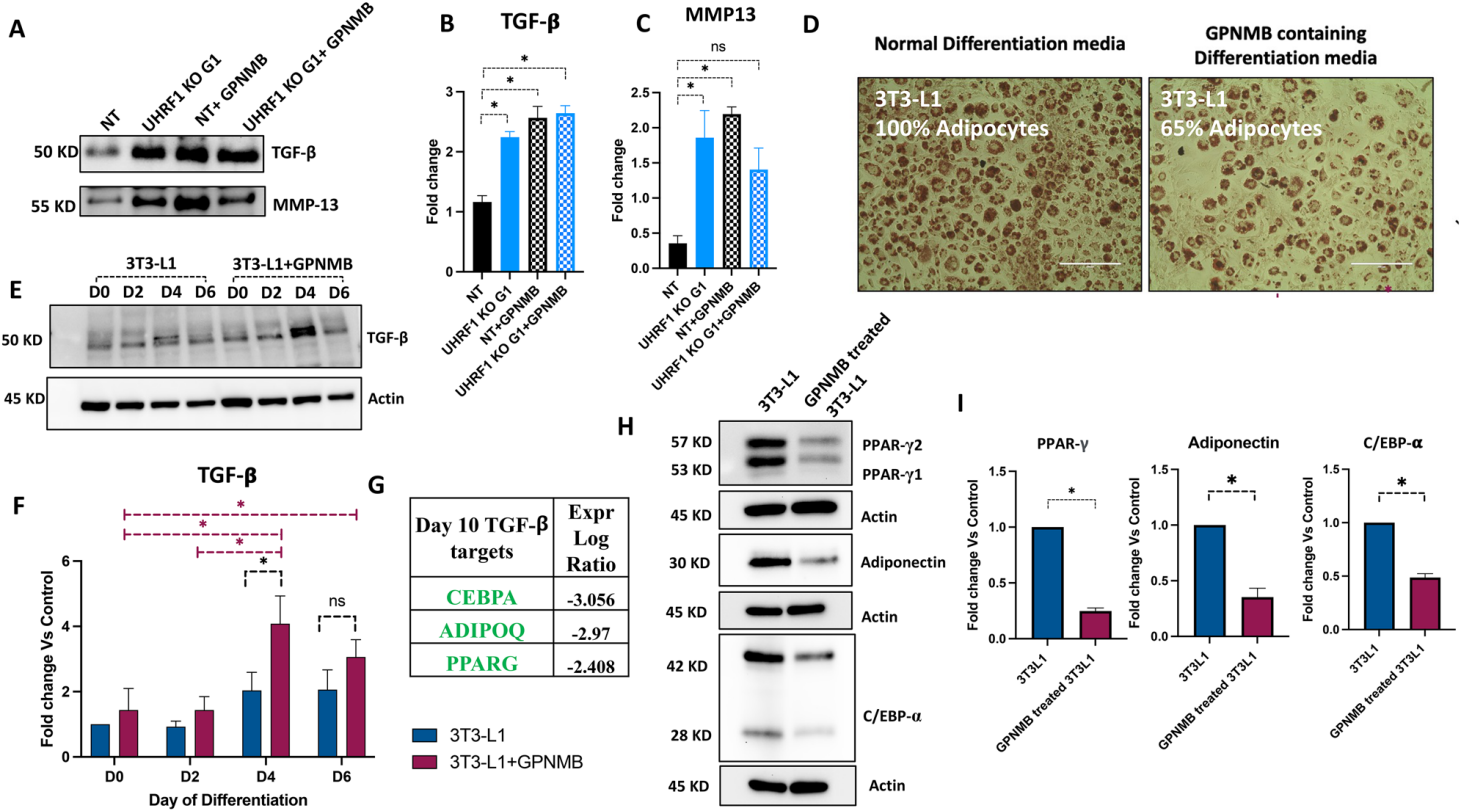
Recombinant GPNMB induces TGF-β and suppresses adipogenesis in 3T3-L1 cells. (**A**–**C**) Western blot analysis of secreted TGF-β and MMP13 in conditioned media collected from NT, UHRF1 KO G1, GPNMB-treated NT, and GPNMB-treated UHRF1 KO G1 cells and its quantification data. (**D**) Oil Red O images of control and recombinant GPNMB (100 ng/ml) treated 3T3-L1 cells on day 8 post-induced 3T3-L1 cells (scale bar, 200 μm). (**E**, **F**) Western blot analysis showing induction of TGF-β in recombinant GPNMB-treated 3T3-L1 during adipocyte differentiation and its quantification graph. (**G**) Expression log ratio of TGF-β targets in adipocyte data set. (**H**) Western blot analysis of adipogenic markers such as PPAR-γ, C/EBP-α, and Adiponectin in control and GPNMB-treated 3T3-L1 cells. (**I**) Western blot quantification graph of PPAR-γ, C/EBP-α, and Adiponectin in control and GPNMB-treated 3T3-L1 cells. Data are mean ± SEM of 3 independent experiments. Statistical analyses were performed using ordinary one-way ANOVA, *P < 0.05.

Accumulating evidence suggests that GPNMB and TGF-β expression are interrelated^41,45^. To confirm this, we treated 3T3-L1 cells with recombinant GPNMB and analyzed the expression of TGF-β. First, we confirmed the non-cytotoxicity of recombinant GPNMB by treating cells with different combinations of recombinant GPNMB and calf serum. No cytotoxicity occurred with GPNMB treatment even at the highest tested concentration (Supplementary Fig. 7). Based on these results and previous reports^44,46–48^, we selected 100 ng/ml of recombinant GPNMB for further experiments. We collected conditioned media from NT and UHRF1 KO G1 preadipocytes after incubating them in serum-free media ± GPNMB for 24 h. As shown in Fig. 6A, treatment with recombinant GPNMB enhanced TGF-β secretion in NT compared to untreated NT. By contrast, TGF-β was present in both untreated and treated UHRF1 KO G1 conditioned media (Fig. 6A). GPNMB has been reported for upregulating MMP13^45,49^. Consistent with these reports, recombinant GPNMB treatment in NT enhanced MMP13 secretion. However, the secretion of MMP13 in UHRF1 KO G1 decreased when we added the recombinant GPNMB, which warrants further experiments to understand the mechanism (Fig. 6A–C). In summary, these results show that recombinant GPNMB treatment in NT cells can induce the secretion of TGF-β and MMP13.

Next, to investigate whether GPNMB hinders adipogenesis, we differentiated 3T3-L1 cells in the presence of 100 ng/ml recombinant GPNMB in differentiation and post-differentiation medium. As shown in Fig. 6D, we observed reduced adipogenesis in GPNMB-treated cells on day 8, with a 35% reduction in adipocytes by Oil Red O staining. Western blot analysis of cell lysates from 3T3-L1 cells treated with GPNMB revealed significant induction of TGF-β on day 4 in treated cells relative to untreated cells (Fig. 6E, F). We also observed significant downregulation of adipocyte markers like PPAR-γ, adiponectin, and CEBP-α in GPNMB-treated cells (Fig. 6G–I). Collectively, these data demonstrate that GPNMB inhibits adipocyte differentiation by suppressing adipocyte-specific markers through TGF-β activation.

### Overexpression of UHRF1 downregulates levels of pro-fibrotic proteins and promotes adipogenesis in human cells

To support our knockout and knockdown studies in 3T3-L1 mouse preadipocytes and to investigate if UHRF1 regulated GPNMB and TGF-β (and its targets) levels in human cells, we performed experiments in HEK293T cells and SW872 preadipocytes after overexpressing recombinant GFP-tagged UHRF1 (UHRF1-GFP) or GFP alone (GFP Control) (see “Methods”).

**Figure 7:**
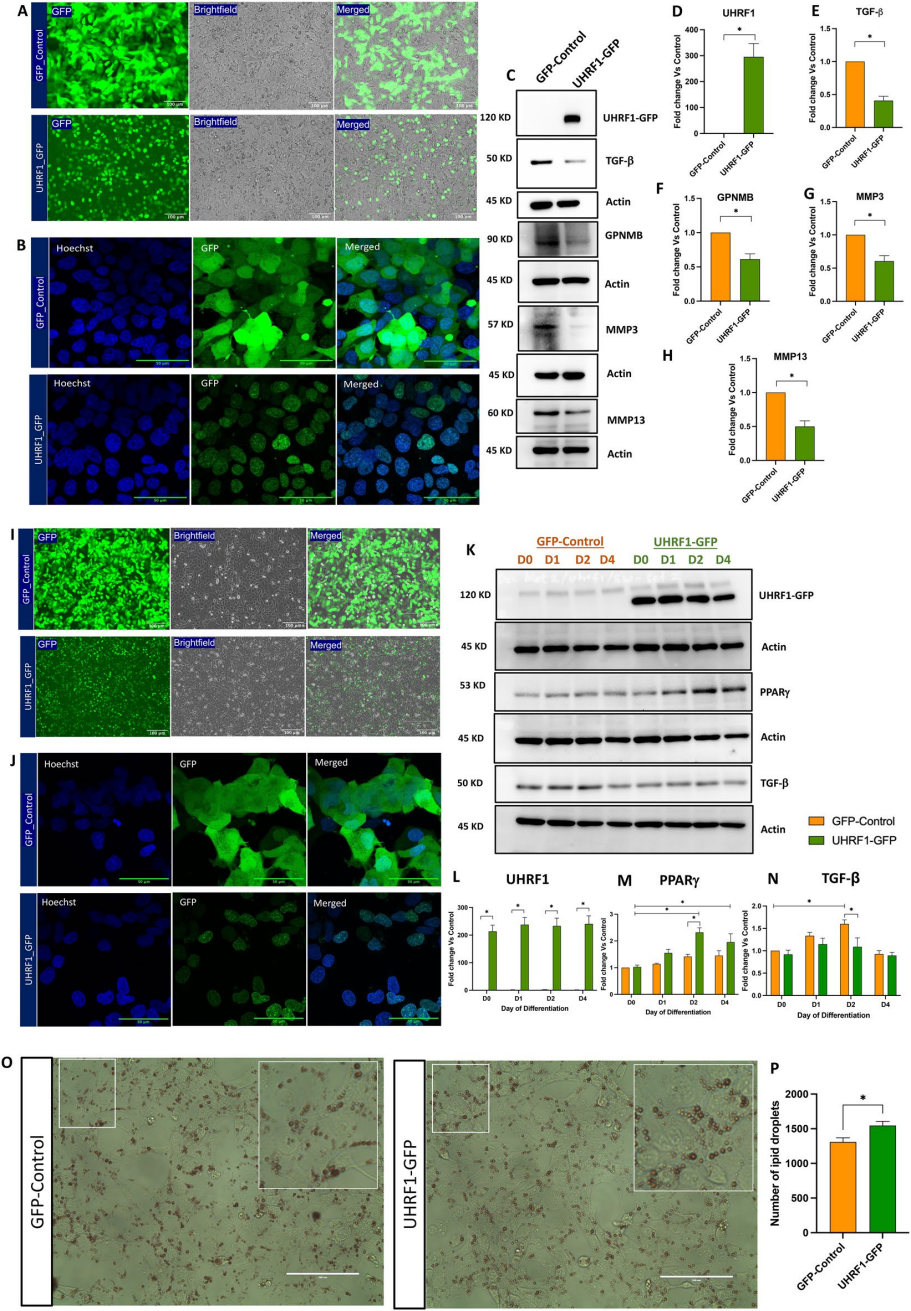
Overexpression of UHRF1 reverses the phenotype observed in UHRF1-depleted cells. (**A**) Representative fluorescence and brightfield microscopy images of lentivirus-mediated expression of GFP control or UHRF1-GFP in HEK293T cells (Scale bar, 100 μm). GFP-positive cells in the total cell population are shown in the merged image. (**B**) Representative confocal microscopy images of GFP control and UHRF1-GFP in HEK293T cells. Nuclei were stained with Hoechst (blue). Scale bar, 50 μm. (**C**–**H**) Representative western blot data and quantification of changes in the expression levels of UHRF1-GFP, TGF-β, GPNMB, MMP3 and MMP13 levels in UHRF1-GFP HEK293T cells *versus* GFP control. Target protein levels were normalized to Actin using ImageJ software. (**I**) Fluorescence and brightfield microscopy images of lentivirus-mediated expression of GFP control and UHRF1-GFP in SW872 cells (Scale bar, 100 μm). GFP-positive cells in the total cell population are shown in the merged image. (**J**) Representative confocal microscopy images of GFP control and UHRF1-GFP in SW872 cells. Nuclei were stained with Hoechst (blue). Scale bar, 50 μm. (**K**–**N**) Representative western blot data and quantification of changes in the expression levels of UHRF1-GFP, PPAR-γ and TGF-β in UHRF1-GFP SW872 cells *versus* GFP control. Target protein levels were normalized to Actin using ImageJ software. (**O**, **P**) Oil Red O staining images of oleic acid (50 µM)-induced differentiation of SW872 cells overexpressing GFP control or UHRF1-GFP on day 4 post-differentiation. Scale bar 100 μm. (**P**) Bar graph showing the total number of lipid droplets present in the entire 40 × field Oil Red O images of GFP control and UHRF1-GFP. Data are mean ± SEM of 3 independent experiments. Statistical analyses were performed using ordinary one-way ANOVA, *P < 0.05.

In HEK293T cells, fluorescence microscopy revealed striking localization of UHRF1-GFP to the nucleus, in sharp contrast to the diffuse localization of the GFP Control (Fig. 7A). Colocalization studies using confocal microscopy after Hoechst-labelling of nuclei confirmed the nuclear localization pattern of UHRF1-GFP (Fig. 7B).We next confirmed a robust increase in UHRF1 protein levels in cells overexpressing UHRF1-GFP *versus* GFP Control. Then, we investigated if UHRF1 overexpression altered the expression levels of GPNMB, TGF-β, and its targets MMP3 and MMP13. Results demonstrated that overexpression of UHRF1 significantly reduced the expression levels of these proteins (Fig. 7C–H).

Since depletion of UHRF1 in 3T3-L1 preadipocytes impaired adipogenesis, we analysed the effect of UHRF1 overexpression on the adipogenic potential of SW872 cells—a widely used human preadipocyte cell-line^7,50^. In line with observations made in HEK293T cells, fluorescence microscopy revealed striking localization of UHRF1-GFP to the nucleus in SW872 cells, in sharp contrast to the diffuse localization of the GFP Control (Fig. 7I). Colocalization studies using confocal microscopy after Hoechst-labelling of nuclei confirmed the nuclear localization pattern of UHRF1-GFP (Fig. 7J). Next, we compared the adipogenic potential of SW872 cells overexpressing UHRF1 with GFP control cells. Cells were differentiated using 50 μm oleic acid and analysed for the expression of PPAR-γ at different time points during differentiation (day 0, 1, 2, and 4). Western blot results showed a significant induction of PPARγ in SW872 cells overexpressing UHRF1 *versus* GFP controls 2 days post-differentiation (Fig. 7K–M). To assess changes in TGF-β levels after UHRF1 overexpression, we analysed its levels during differentiation. Interestingly, we observed early suppression of TGF-β expression in UHRF1-GFP cells (Fig. 7K, N), which supports our observations in 3T3-L1 mouse preadipocytes (see Fig. 5C). Further, Oil Red O staining of differentiated SW872 cells 4 days post-differentiation demonstrated a significant increase in the number of lipid droplets in UHRF1-overexpressing cells than in GFP Control (Fig. 7O, P). Overall, these results suggest that UHRF1 promotes adipogenesis and negatively regulates GPNMB and TGF-β levels in human cells.

## Discussion

Using transcriptomics approaches and downstream analyses, we identified UHRF1 as a key regulatory factor in adipogenesis. We demonstrated that both UHRF1 knockout and UHRF1 knockdown in 3T3-L1 preadipocytes leads to impaired adipocyte differentiation and induction of TGF-β signaling, fibrosis, and inflammation. We also found that depletion of UHRF1 significantly decreased the expression of adipocyte markers such as PPAR-γ and C/EBP-α, and increased expression of GPNMB and TGF-β. Recombinant GPNMB treatment increased TGF-β expression in 3T3-L1 cells and suppressed adipocyte differentiation, suggesting adipogenesis modulation in UHRF1-null cells. In addition, UHRF1 overexpression studies in human cells demonstrated that UHRF1 negatively regulated the expression levels of GPNMB and TGF-β in HEK293T cells and enhanced the adipogenic potential of SW872 preadipocytes.

UHRF1 is an epigenetic adapter protein involved in DNA replication and cell proliferation^51,52^. Consistent with previous reports, we found upregulation of UHRF1 in the MCE phase of NT and shScramble cells. The gradual downregulation of UHRF1 during later stages of differentiation has been reported before^53^. By comparison, UHRF1-depleted cells showed impaired MCE and reduced lipid accumulation. Inagaki et al. showed impaired MCE followed by adipogenesis suppression in FBXL10-overexpressing 3T3-L1 cells. FBXL10 epigenetically represses UHRF1, Cdk1, and PPAR-γ by recruiting Polycomb repressive complex 1 (PRC1) complex, resulting in G1/S and G2/M phase cell cycle arrest leading to inhibition of terminal differentiation^54^. Adipogenesis differentiation induction triggers immediate expression of several transcription factors, including C/EBP- β, whose activation and acquisition of DNA-binding activity is restricted to the MCE of the adipogenesis phase^55^. Reports have shown that C/EBP-β expression is a prerequisite for MCE in the adipocyte-differentiation program^55^ and is then followed by binding C/EBP-β in proximity to the promoters of PPAR-γ and C/EBP-α to upregulate their expression, which leads to the activation of multiple downstream adipocyte-specific genes required for triglyceride synthesis and lipid droplet accumulation^56^. Here, the immediate induction of C/EBP-β expression in UHRF1-depleted cells was similar to that of the control; yet MCE and downstream essential adipogenic genes such as PPAR-γ and C/EBP-*α* were differentially suppressed upon UHRF1 deletion, suggesting that UHRF1 plays a functional role in initiating the expression of these critical genes. Altogether, these results indicate that UHRF1 is required for MCE and preadipocyte differentiation.

Upregulation of GPNMB in UHRF1 KO neural stem cells^57^ and UHRF1 KO cancer cells^58^ have been reported before. Therefore, our results of GPNMB upregulation in both UHRF1 KO preadipocytes and UHRF1 KO adipocytes match with previous reports. The ECD of GPNMB can undergo ectodomain shedding by the action of proteases^44^. However, murine GPNMB secretion retained the C terminus, indicating non-ectodomain shedding from the plasma membrane^32^. Murine GPNMB is mainly localized in lysosomes and endosomes^36^. Consistent with these reports, our immunofluorescence results showed abundant expression of GPNMB in the cytoplasm of UHRF1 KO cells. In addition, the secretion of GPNMB in the conditioned media of UHRF1 KO demonstrates that it can function as a signaling molecule; however, the mechanism remains to be explained.

The downregulation of canonical pathways—including oxidative phosphorylation, amino acid degradation, TCA cycle, beta-oxidation, glycolysis, and cholesterol biosynthesis—in UHRF1 KO cells suggests a possible functional role for UHRF1 in pathways related to adipogenesis. Upregulation of canonical pathways such as fibrosis, TGF-β signaling, cell adhesion, and ECM accumulation^59,60^ indicates dysfunctional adipocyte molecular signatures. We found upregulation of fibrotic markers such as PDGFC, TGFB3, TNC, FN1, CCN2, EDN1, SMAD1, and inflammatory markers such as IL1A, IL6, CCN1, BMP4, and BMP2 in the UHRF1-null adipocyte dataset. Consistently, we found increased expression of TGF-β and MMPs at the protein level, and transcriptional upregulation of several fibrosis and inflammatory markers including COL, MMPs, IL-6, and MCP-1. This aligns with the increased inflammatory markers observed in transgenic mice with UHRF1-deficiency in macrophages^20^ and increased ECM and cell adhesion-related genes in UHRF1-deficient Sertoli cells^61^.

TGF-β regulation is highly complex, and its activity is regulated by extracellular activation and glycoproteins^62^. In Neural Stem Cells (NSCs), TGF-β was identified as an upstream activator of GPNMB; however, the elevation of GPNMB in these cells resulted in further enhancement of TGF-β1 signaling and inhibition of oligodendrocytes differentiation^63^. GPNMB binding to the syndecan-4 (SD-4) receptor on activated T cells causes transforming growth factor-β (TGF-β) to become trapped on the cell surface^64,65^. These results suggest that GPNMB and TGF-β might interact in a feedback manner. Consistent with these reports, our western blot results showed induction of TGF-β during the late phase of differentiation in UHRF1 KO cells and a synergistic enhancement of TGF-β expression with recombinant GPNMB treatment in non-infected cells. Our data generated in HEK293T and SW872 cells overexpressing UHRF1 support our findings in 3T3-L1 preadipocytes and suggest cell-type independent regulation of these proteins by UHRF1.

Different mechanisms are reportedly involved in TGF-β signaling, including increased TGF-β deposition at the ECM and its activation by proteases^66^. In support, we found transcriptional upregulation of MMP3 and MMP13 in UHRF1-depleted cells. Interestingly, MMP13 overexpression in human osteoarthritic cartilage is reported to cause SMAD-independent TGF-β signaling pathway^67^. Given that GPNMB activates MMPs in different cell lines, including fibroblasts^68,69^, we speculate that MMP13 upregulation by GPNMB might lead to the expression of TGF-β. Alternatively, TGF-β signaling is tightly regulated by E3 ligases, such as Smurfs, Skp2, Arkadia, Ectodermin, and WWP1. E3 ubiquitin tag on TGF-β type 1 receptor (TGF-β R1) is followed by ubiquitin-mediated proteolysis^70–72^. Given the E3 ubiquitin ligase role of UHRF1^73^, it is rational to connect the upregulated TGF-β signaling to UHRF1-depletion in cells.

TGF-β inhibits adipogenesis by upregulating the expression of collagen, fibronectin, and ECM coding genes^74,75^ and suppressing genes involved in adipogenesis^75^. Considering the recent reports of fibrosis as a multifactorial condition involving multiple factors, such as TGF-β, TNF-α, interleukins and chemokines ligand-receptor superfamilies^76^, we suggest that UHRF1 may have a role in fibrosis by regulating these factors. Several epigenetic modifications, including changes in DNA methylation and histone modification, have recently been shown to be involved in fibrosis progression^77^. In support, in UHRF1-deficient Sertoli cells, the upregulation of many ECM-related genes, such as TIMP1, SPP1, and TRF, was mediated through hypomethylation in their genomes, which points towards the possible role of UHRF1 in regulating ECM and cell adhesion-related genes^58^. A study in hepatic stellate cells also showed that inhibition of DNMT1 caused Smad7 activation, which led to increased collagen I production^78^. Overall, considerable evidence suggests that UHRF1 could function as a critical regulator in fibrosis.

Consistent with the existing literature, along with TGF-β expression, we found transcriptional upregulation of collagens and fibronectin and suppression of adipocyte markers such as PPAR-γ, CEBP-α, and adiponectin in UHRF1-depleted cells. Elevated levels of GPNMB have been reported in DIO and inflammation^43,79^. Patients with non-alcoholic steatohepatitis (NASH), one of the significant comorbidities of diabetes and obesity, show a higher level of GPNMB in their plasma^79^. GPNMB-ECD overexpressing mice displayed severe insulin resistance, in which treatment with GPNMB-neutralizing antibodies significantly improved metabolic parameters and insulin sensitization^30^. Altogether, these previous results highlight that UHRF1 can be a novel target for limiting fibrosis and insulin resistance in adipocytes.

Several studies have shown the involvement of epigenetic mechanisms in the transcriptional regulation of GPNMB. GPNMB is one of the target genes for enhancement of zeste homolog 2 (EZH2), a histone methyltransferase. EZH2 represses the GPNMB promoter by mediating H3K27 methylation^80^. Interestingly, UHRF1 expression positively correlates to EZH2 expression^81,82^; therefore, the induction of GPNMB in UHRF-depleted cells could result from transcriptional reprogramming. A similar mechanism may underlie the reduction in GPNMB levels in HEK293T cells after overexpression of UHRF1, especially considering the nuclear localization pattern that was observed for recombinant UHRF1 in this cell-type. Studies have also reported microphthalmia-associated transcription factor (MITF) based GPNMB transcriptional regulation^31,83^. MITF is a master regulator of the lysosome, autophagy, melanocyte proliferation, and differentiation^84,85^. GPNMB is upregulated in stressed lysosomes in different disease conditions^35,37^, such as Gaucher disease (GD)^86^, and the condition is associated with insulin resistance, low circulating adiponectin^87^. These studies show that lysosomal stress can trigger GPNMB expression and is accompanied by insulin resistance. Consistent with these reports, in UHRF1-lacking cells, we found transcriptomic upregulation of lysosomal markers, including LAMP1, ATP6V0A4, MCOLN1, CTSD, AGA, GBA, and others, increased MITF, and reduced GLUT4 expression.

GPNMB is also known as osteoactivin for its potent osteogenic inductive activity^88^. MSCs can undergo differentiation into mutually exclusive lineages such as adipocytes and osteocytes^89^. Moreover, the master regulator of osteogenesis, Runx-related transcription factor 2 (RUNX2), and the master regulator of adipogenesis, PPAR-γ, repress each other’s functions^90^. Interestingly, in C2C12 myogenic cells, TGF-β mediated RUNX2 and ALP expression are reported^91^, indicating enhancement of osteogenesis in TGF-β activated cells. GPNMB-mediated enhancement of focal adhesion, ECM interactions^39^, TGF-β signaling^39,92,93^, osteogenesis, osteoclast differentiation^39^, and PI3K-AKT signaling^40^ were previously reported. Consistent with these reports, we found upregulation of RUNX2, calcium signaling pathway, osteoclast differentiation, ECM-receptor interaction, focal adhesion, and fibrosis in UHRF1 KO datasets, suggesting that the microenvironment might be favorable for osteogenesis, restricting adipogenesis.

In conclusion, this study has shown that UHRF1 is a crucial factor for adipogenic differentiation, and its expression in the early phase is coupled with the induction of adipogenic markers PPAR-γ and C/EBP-α. Our data also indicate that UHRF1 is a negative regulator of TGF-β and GPNMB, suggesting an essential role for UHRF1 in determining adipocyte functional characteristics. This study is the first to report that UHRF1 modulates adipogenesis through GPNMB and TGF-β activation, and it highlights the significance of studying UHRF1 and its targets as a potential therapeutic approach to obesity preventive healthcare.

## Materials and methods

### Cell culture

Mouse 3T3-L1 preadipocytes (#SP-L1-F, Zen-Bio, Durham, NC, USA) were cultured in DMEM high-glucose medium (DMEM, catalog no.12430054, Invitrogen, Carlsbad, CA, USA), supplemented with 10% calf serum (Catalog no.16170078, Invitrogen) and 1% penicillin–streptomycin (Catalog no.15240002, Invitrogen) at 37 °C in a humidified incubator containing 5% CO_2_^7,8^_._ Human SW872 preadipocytes (ATCC) were cultured in DMEM/F12 medium supplemented with 8% fetal bovine serum (FBS) (catalog no. AlphaFBS-HI, Alphabioregen, Boston, MA, USA), 1% penicillin–streptomycin and 15 mM Hepes. HEK293T viral packaging cells were purchased from ATCC and maintained in high-glucose DMEM supplemented with 10% FBS and 1% penicillin–streptomycin antibiotics. Cells were sub-cultured at 70–80% confluence.

### Generation of UHRF1 knockout cells by clustered regularly interspaced short palindromic repeats/CRISPR associated protein 9 (CRISPR/Cas9) technology

To generate UHRF1 Knock out cells, guide RNA sequence cloned into lentiCrispr v2 plasmid targeting Uhrf1 and Control NT was purchased from Genscript (Piscataway, NJ, USA) and generated as described previously^94^. Two guide RNA sequences were used, and the cells generated were termed UHRF1 KO gRNA 1 (UHRF1 KO G1) and UHRF1 KO gRNA 2 (UHRF1 KO G2). The gRNA target sequences were: UHRF1 gRNA 1: 5′-TCACAGTGCGAGCACGAGCA-3′, UHRF1 gRNA-2: 5′-GACTCTGGCGCACGAGCAGC-3′ and NT gRNA: 5′-GCTTTCACGGAGGTTCGACG-3′. Briefly, HEK293T cells were co-transfected with packaging, envelope (pVSVg and psPAX2), and transfer plasmids using lipofectamine 2000 following the manufacturer’s instructions. After 6 h, the medium was changed to DMEM supplemented with 10% FBS and incubated for 72 h. Media were collected and centrifuged at 1500 rpm, 4^◦^C for 5 min, and the supernatant was filtered through 0.2 μm membrane filters (Millipore, Burlington, MA, USA) to collect the lentiviruses containing media. Collected lentiviruses were used to infect early passage 3T3-L1 preadipocytes using 4 μg/ml polybrene (Santacruz Biotechnology, Dallas, TX, USA). After 48 h incubation, cells were trypsinized and sub-cultured in 2 μg/ml puromycin (Life Technologies, Carlsbad, CA, USA) containing media for 3 more passages followed by Uhrf1 gene knockout validation by Western blot analysis of UHRF1 protein with Actin as the loading control.

### Generation of UHRF1 knockdown cells by shRNA targeted Uhrf1 silencing in 3T3-L1 preadipocytes

UHRF1 knockdown in 3T3-L1 preadipocytes was achieved using a lentiviral vector (MISSION^®^ shRNA plasmid DNA, Sigma-Aldrich) expressing shRNA targeting mouse UHRF1 mRNA. Two constructs were used, and the cells generated were shUHRF1 Construct1 (shUHRF1 C1) and shUHRF1 Construct2 (shUHRF1 C2). shRNA sequence with no homology in the mouse (shScramble) was used as a control. The sequences were as follows: shUHRF1 C1: 5′-CCGGCTGTAGCTCCAGTGCCGTTAACTCGAGTTAACGGCACTGGAGCTACAGTTTTTG-3′, shUHRF1 C2: 5′-CCGGCACACTCTTCGATTATGATCTCGAG ATCATAATCGAAGAGTGTGTGTTTTTG-3′. A non-sense RNAi sequence (shScramble) with no homology to the mouse genome was used as the control^7,95^. In brief, 293 T cells at 60% confluence was co-transfected with shRNA lentiviral plasmid, packaging, and envelope plasmids (pVSVg and psPAX2) using lipofectamine 2000 (Invitrogen) according to manufacturer’s instructions. After 72 h of incubation, media were collected and centrifuged to remove the debris. The supernatant containing generated ShUHRF1 and ShScramble viral particles was used to infect 70% confluent 3T3-L1 preadipocytes with 4 μg/ml polybrene. After 48 h incubation, transduced cells were sub-cultured in 2 μg/ml puromycin-containing media to select UHRF1-depleted cells.

### Lentivirus-mediated overexpression of UHRF1

GFP Control (PS100093) and GFP-tagged UHRF1 (MR226251L4) plasmids were purchased from Origene. To generate lentiviral particles, we used both 2^nd^ and 3^rd^ generation packaging plasmids and both approaches were successful. Briefly, HEK293T packaging cells were co-transfected in serum-free medium with the transfer plasmids (6–10 µg GFP or UHRF1-GFP) and 6–10 µg packaging plasmids using lipofectamine for 6 h, followed by switch to antibiotic-free 10% FBS-containing medium. 72 h later, the viral supernatant was collected, centrifuged, filtered using 0.22 µm filter, aliquoted, and stored at − 80 °C. To generate stable HEK293T and SW872 cells overexpressing GFP or UHRF1-GFP, we used the method described in the previous section. Stable HEK293T and SW872 cells were selected using puromycin concentrations of 1 μg/ml and 0.5 μg/ml, respectively. Because both plasmids express GFP, successful transduction and enrichment were confirmed using fluorescence microscopy.

### Adipocyte differentiation protocol

Cells were seeded in 6-well plates at a density of 200,000 cells/well in DMEM media and cultured to confluence. Two days post-confluence, cells were induced to differentiate using an adipogenic differentiation cocktail containing 500 μM IBMX (Sigma), 1 μM dexamethasone (Sigma), 10 μg/ml insulin (Sigma) with 10% FBS and 1% antibiotic. After 2 days, the media was replaced with post-differentiation media containing 10 μg/ml insulin, 10% FBS, and 1% antibiotic. Subsequently, media was changed every 2 days until the complete differentiation of control cells by day 10 post-induction. Cells were collected for RNA and protein on days 0, 1, 2, 4, 6, 8, and 10 for further analysis (Supplementary Fig. 8).

### Growth curve

Cells were plated in 6-well plates at a density of 50,000 cells/well, and at different post-plating time points, cells were trypsinized, and pellets were resuspended in media. 1:1 trypan blue dye and cell suspension volume/volume ratio were mixed and loaded to cell counting slides, and cell numbers were recorded using a TC10 automated cell counter (Bio-Rad).

### Oil red O staining

Day 10 differentiated cells were washed with PBS and fixed with 4% paraformaldehyde (PFA) for 15 min at room temperature. After rinsing with PBS, cells were treated with 60% isopropanol in PBS for 1 min, followed by incubation with 60% of Oil Red O stain (Sigma-Aldrich) in PBS for 1 h at room temperature. For the Oil Red O stock preparation, 500 mg Oil Red O powder was dissolved in 100 ml of 99% isopropyl alcohol. After staining, cells were washed with water, followed by counterstaining of nuclei with hematoxylin for 1 min. After a final wash with water, cells were mounted in 70% glycerol and examined under a digital EVOS™ microscope with an AMG camera to observe lipid droplets.

### Western blot

Cells were rinsed with ice-cold PBS, and lysates were prepared in RIPA buffer (Invitrogen) containing protease inhibitor cocktail (Sigma), 1 mM sodium orthovanadate (Sigma), 5 mM benzamidine (Sigma), 1 mM phenylmethylsulfonyl fluoride (PMSF) (Sigma), 10 mM sodium fluoride (Sigma), 20 µg/ml calpain inhibitor (Sigma), 3 mM trichostatin A (InvivoGen) and 5 mM nicotinamide (Sigma). Lysates were centrifuged at 15000 g at 4 °C for 10 min, the supernatant was collected, and the protein concentration was measured by DC assay (Bio-Rad) using a CLARIOstar microplate reader (BMG LABTECH, Offenburg, Germany). 30 μg of protein was subjected to sodium dodecyl sulfate–polyacrylamide gel electrophoresis (SDS-PAGE) separation and transferred to a polyvinylidene fluoride (PVDF) membrane (Bio-Rad). A molecular weight marker (Thermo Fisher Scientific) was included to confirm the molecular weight of the protein. Membranes were then blocked using 4% bovine serum albumin (BSA) (Sigma) for 1 h at room temperature, followed by overnight incubation with the primary antibody at 4 °C. Antibodies used were: Uhrf1 (D6G8E, Rabbit mAb#12,387, cell signaling Techno.), β-Actin (8H10D10, Mouse mAb#3700, cell signaling Techno.), C/EBPβ (Rabbit, ab32358, Abcam), PPAR-γ (Rabbit#2443, Cell Signaling Techno.), C/EBPα (Rabbit #2295, Cell Signaling Techno.), Glut 4 ((1F8) Mouse Ab#2213, Cell Signaling Techno.), GPNMB ((E7U1Z) Rabbit mAb#90205, Cell Signaling Techno.), Adiponectin (Rabbit, C45B10, Cell signaling Techno.), TGF-β (Rabbit, 3711S, Cell signaling Techno.), MMP13 (ab39012, RRID: AB_776416, Abcam), Anti-rabbit IgG (7074, RRID: AB_2099233, Cell Signaling Techno.), and Anti-mouse IgG (7076, RRID: AB_330924, Cell Signaling Techno.). The next day, after washing with tris-buffered saline tween (TBST) 3 times, membranes were incubated with secondary antibody for 1 h at room temperature. The Chemiluminescent ECL reagent, SuperSignal West Dura Extended Duration Substrate (Thermo Fisher Scientific), was used to detect the protein bands using the ChemiDoc™ MP imaging system (Bio-Rad). Blots were stripped and reprobed for β-Actin (8H10D10, Mouse mAb #3700, Cell Signaling Techno.) to quantitate the expression of target protein against the loading control. Band intensities were quantified using ImageJ software https://imagej.nih.gov. All the original raw blots are included in the Supplementary Fig. 10.

### Conditioned media experiment

Cells were seeded in a 10 cm dish, and upon confluence, cells were washed with DMEM media containing 1% antibiotic to remove residual serum. Then cells were incubated with 3.5 ml DMEM media with 1% antibiotic and 1% ITS (Insulin-Transferrin-Selenium) for 24 h. The next day, conditioned media were collected and filtered through a 0.2-μm filter to remove cellular debris and aliquoted in Eppendorf tubes and stored at − 80 °C.

### RNA extraction and real-time PCR

Total RNA was extracted from Qiazol lysed cells using miRNeasy Mini Kit (#217004, QIAGEN) per the manufacturer’s instructions. DNase1 (#79254, QIAGEN) was added to the filters and incubated for 15 min at room temperature to enhance the RNA purity by digesting contaminant DNA. Quantification of eluted RNA was determined using a Nanodrop spectrophotometer (ThermoFisher Scientific). 1 μg of RNA was reverse transcribed to cDNA using High-Capacity RNA-to-cDNA Kit ((#4387406, Applied Biosystems, Foster City, CA, USA). cDNA was then subjected to Quantitative PCR (qPCR) to analyze the expression of selected genes. SYBR Select Master Mix (Applied Biosystems) and QuantStudio™ 6 Flex Real-Time PCR System (Applied Biosystems) were used to perform qPCR according to the manufacturer’s instructions. The protocol used was as follows: denaturation (50 °C, 20 s; 95 °C, 10 min) followed by 40 amplification cycles (95 °C, 15 s; 60 °C, 1 min) and melting curve analysis steps (95 °C, 15 s; 60 °C, 1 min; 95 °C, 30 s; 60 °C, 15 s). Gene expression levels were normalized relative to the housekeeping gene NONO expression level and quantified by the comparative threshold cycle (ΔΔCT) method. Primer sequence details are in Supplementary table 1.

### Transcriptomics and *IPA*

RNA-sequencing was performed on the total RNA extracted from the NT and UHRF1 KO G1 at two-time points, day 0 and day 10 of adipocyte differentiation. Three biological replicates were used for data generation. The WCM-Q Genomics Core Facility prepared the cDNA library and sequencing. The WCM-Q Bioinformatics Core Facility performed the read-mapping. 100 bp paired reads were mapped to the mouse reference genome obtained from GENCODE-built M10, GRCm38 assembly using Tophat2 (version 2.1.0). Ensembl85 gene annotations were used. The gene expression level was quantified by the feature counts function from Rsubread (version 1.22.3), with Bioconductor Package in R (version 3.3.2). All the read counts from conditions were combined into a data matrix. Based on gene identifiers, aligned reads were quantified and genes with at least one read were selected. The counts were normalized using the default method (Relative Log Expression) in DESeq2 (version 1.12.4)^96^. Then the DEGs between groups were identified using the Wald test from DESeq2 algorithms. To select DEGs, the significance threshold was set at a *P*-value of 0.05. IPA software (IPA, Ingenuity Systems, http://www.ingenuity.com) was used to identify differentially altered signaling pathways. The raw transcriptomics dataset is included in the supplementary file.

### Immunofluorescence

Cells were cultured on glass-coverslip bottom plates and fixed with 4% paraformaldehyde for 20 min then permeabilized using 0.1% Triton X-100 in PBS for 10 min at room temperature. After 3 washes with PBS, 5% BSA was added and incubated for 30 min, followed by overnight incubation with the GPNMB primary antibody at 4 °C. The next day, the cells were washed with PBS and incubated with Alexa Fluor 594/488-conjugated anti-rabbit secondary antibodies (1:300) (Jackson Immuno Research, West Grove, PA, USA) for one hr at room temperature. Nuclei of cells were stained with DAP1 and detected cells under a Confocal microscope. The intensity of immunofluorescence-positive cells was quantified by Image J software (NIH).

### Confocal microscopy

Cells expressing GFP Control or UHRF1-GFP were plated in 1% w/v gelatin-coated glass-bottom 6-well plates (MatTek, #P06G-1.5-10-F) at a density of 500 × 10^3^ cells/well and imaged 2 days post-plating. On the day, cells were incubated with 2 µg/ml Hoechst 33,342 (#H3570, ThermoFisher Scientific) prepared in Optimem reduced serum medium, and washed 3x (Optimem) prior to imaging. A Carl Zeiss LSM-880 microscope was used to collect the images (63 × objective) and a series of z-sections were collected for each field. Fiji—ImageJ software was used to analyze the images (www.imagej.net/Fiji). The 2-D images were generated by compacting the z-sections using the Z-project tool for presentation.

### Enzyme-Linked Immuno Sorbent Assay (ELISA)

The concentration of GPNMB in NT and Uhrf1 KO preadipocyte conditioned media was measured using GPNMB ELISA (R&D), according to the manufacturer’s instructions. Normalization of conditioned media was done based on the total protein content of cell lysates of preadipocytes.

### Statistical analysis

The results of 3 independent analyses were presented as mean ± standard error of the mean (SEM). GraphPad Prism software was used to plot the data, and groups were compared by ordinary one-way ANOVA, and *P* values < 0.05 were considered as statistically significant.

### Supplementary Information

Supplementary Information.

## Data Availability

All the datasets generated in the current study are available from the corresponding author upon request.
