# Supplementary material for: The E3 ubiquitin-protein ligase UHRF1 promotes adipogenesis and limits fibrosis by suppressing GPNMB-mediated TGF-β signaling
Source: Sci Rep. 2024 May 24;14:11886. doi: 10.1038/s41598-024-62508-y (PMC11126700; doi:10.1038/s41598-024-62508-y)
Supplement: Supplementary file 1 — Supplementary Information. [file 41598_2024_62508_MOESM1_ESM.docx]

**The E3 ubiquitin-protein ligase UHRF1 promotes adipogenesis and limits fibrosis by suppressing GPNMB-mediated TGF-β signaling.**

Muneera Vakayil^1,2^, Aisha Y. Madani^2^, Maha V. Agha^3^, Yasser Majeed^2^, Shahina Hayat^4^, Shameem Yonuskunju^5^, Yasmin Ali Mohamoud^5^, Joel Malek^5^, Karsten Suhre^4^, and Nayef A. Mazloum^2^*

^1^ College of Health & Life Sciences, Hamad Bin Khalifa University, Qatar Foundation, PO Box: 34110, Doha, Qatar.

^2^Department of Microbiology and Immunology, Weill Cornell Medicine-Qatar (WCM-Q), Qatar Foundation, PO Box: 24144, Doha, Qatar.

^3^Translational Research Institute, Academic Health System, Hamad Medical Corporation, PO Box: 3050, Doha, Qatar.

^4^Department of Physiology and Biophysics, Weill Cornell Medicine-Qatar (WCM-Q), Qatar Foundation, PO Box: 24144, Doha, Qatar.

^5^Department of Genetic Medicine, Weill Cornell Medicine-Qatar (WCM-Q), Qatar Foundation, PO Box: 24144, Doha, Qatar.

*Corresponding author: Nayef A. Mazloum (nam2016@qatar-med.cornell.edu)

**Supplementary Figures**

# Supplementary Figure 1: Principal component analysis. PCA plot representing different molecular characteristics in preadipocytes and adipocytes of UHRF1 KO and NT cells.

**Supplementary Figure 2: Summary graph of UHRF1 KO preadipocytes.** Results showing the interaction between the top significant entities and the predicted biological pathways, which include alterations in cell movement and phagocytosis.

**Supplementary Figure 3: Adipocyte markers are transcriptionally downregulated in shUHRF1 C2 cells.** Relative mRNA expression of adipocyte genes, C/EBP-β, PPARγ, and C/EBP-α in shScramble and shUHRF1 C2 at different time points during adipogenic differentiation. mRNA expression of the specific gene was normalized to the expression of control gene, NONO at respective time point, followed by normalization to the day0 sample. Data are mean ± SEM of 3 independent experiments. Statistical analyses were performed using ordinary one-way ANOVA, *P<0.05.

**Supplementary Figure 4: Adipocyte markers are transcriptionally downregulated in UHRF1 KO G1 cells.** Relative mRNA expression of adipocyte genes, C/EBP-β, PPARγ, and C/EBP-α in NT and UHRF1 KO G1 across differentiation after normalized to the control gene, NONO. Data are mean ± SEM of 3 independent experiments. Statistical analyses were performed using ordinary one-way ANOVA, *P<0.05.

**Supplementary Figure 5: Transcriptomics analysis reveal downregulation of hundreds of genes common in UHRF1-null datasets.** A)Venn diagram analysis indicating 281 common downregulated genes in UHRF1 KO preadipocyte and UHRF1 KO adipocyte data set. Expression log ratio of top downregulated common gene Prl2c2 in both data sets. B) The most significant pathways associated to common downregulated genes in UHRF1-null datasets by KEGG pathway analysis [www.kegg.jp/kegg/kegg1.html](http://www.kegg.jp/kegg/kegg1.html). C) The most significant pathways associated to common upregulated genes in UHRF1-null datasets by KEGG pathway analysis.

**Supplementary Figure 6: ADAM10 mRNA is induced in both NT and UHRF1 KO G1** A) Normalization of condition media based on total protein concentration in cell lysates of UHRF1 KO G1 and NT preadipocytes. B) Relative mRNA expression of ADAM10 in UHRF1 KO G1 and NT at different time points during differentiation. Data are mean ± SEM of 3 independent experiments. Statistical analyses were performed using ordinary one-way ANOVA, *P<0.05.

**Supplementary Figure 7: Recombinant GPNMB is non-cytotoxic to 3T3-L1 cells.** A) Cell count of 3T3-L1 cells treated with different combinations of GPNMB and serum concentration. Data are mean ± SEM of 3 independent experiments. Statistical analyses were performed using ordinary one-way ANOVA, *P<0.05.

**Supplementary Figure 8: Schematic representation of adipocyte differentiation protocol.**

**Supplementary Figure 9: Transcriptomics analysis reveals upregulated inflammatory markers in UHRF1 KO G1 adipocytes.**

A) Table showing the number of affected targets of TNF and INFγ, the upstream regulators predicted by IPA analysis at P-value <0.05 and FDR<0.05. Upregulated targets are represented in red colour and downregulated targets are shown in green colour. B) Venn diagram showing the number of common and exclusive targets of TNF and INFγ. C) Table showing the top 25 upregulated and the top 25 downregulated common targets of TNF and INFγ. D-E) Relative mRNA expression of IL-6 in NT, UHRF1 KO G1, shScramble and shUHRF1 C2 at different time points during adipocyte differentiation by qPCR. F-K) qPCR validation of some of the top common targets of TNF and INFγ in NT, UHRF1 KO G1, shScramble and shUHRF1 C2 at different time points of adipocyte differentiation. L-M) Western blot validation of GLUT4 in NT and UHRF1 KO G1 at different time points during adipocyte differentiation and its quantification by normalizing to the loading control, Actin by ImageJ software. N-O) Western blot validation of Leptin in shScramble and shUHRF1 C2 at different time points during adipocyte differentiation and its quantification by normalizing to the loading control, Actin by ImageJ software.

**Supplementary Figure 10: Uncropped Western blot results.** 3 independent experiments were performed, and the image used in the paper is highlighted in red boxes.

Supplementary Figure 1

Supplementary Figure 2


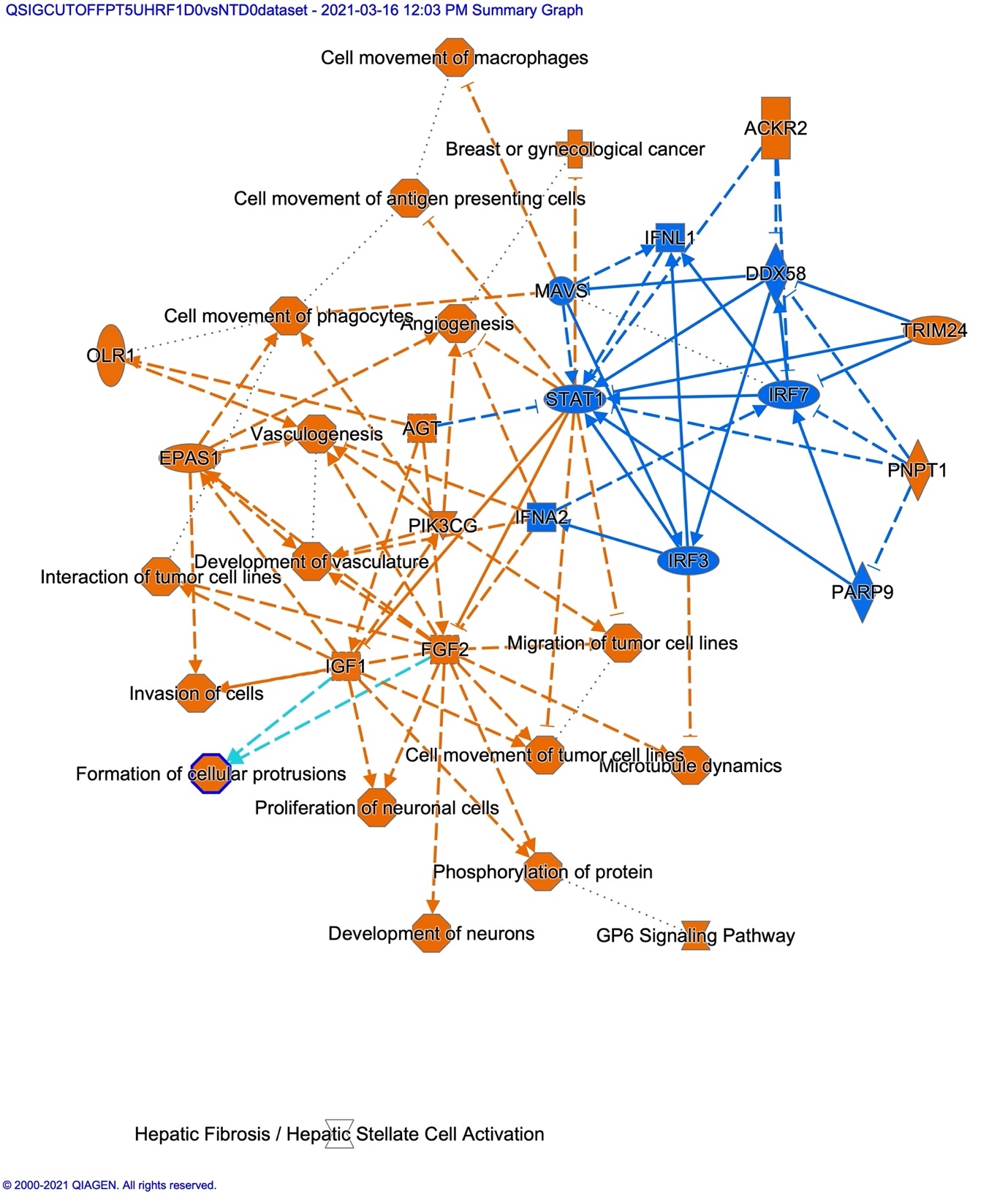


Supplementary Figure 3


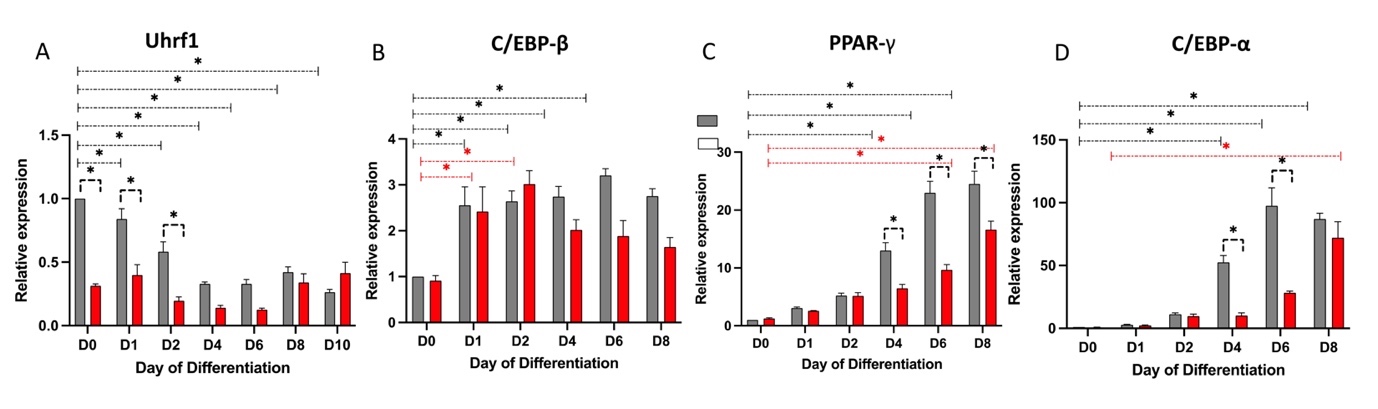


Supplementary Figure 4

Supplementary Figure 5

A

B

C

Supplementary Figure 6

A

B

Supplementary Figure 7

Supplementary Figure 8


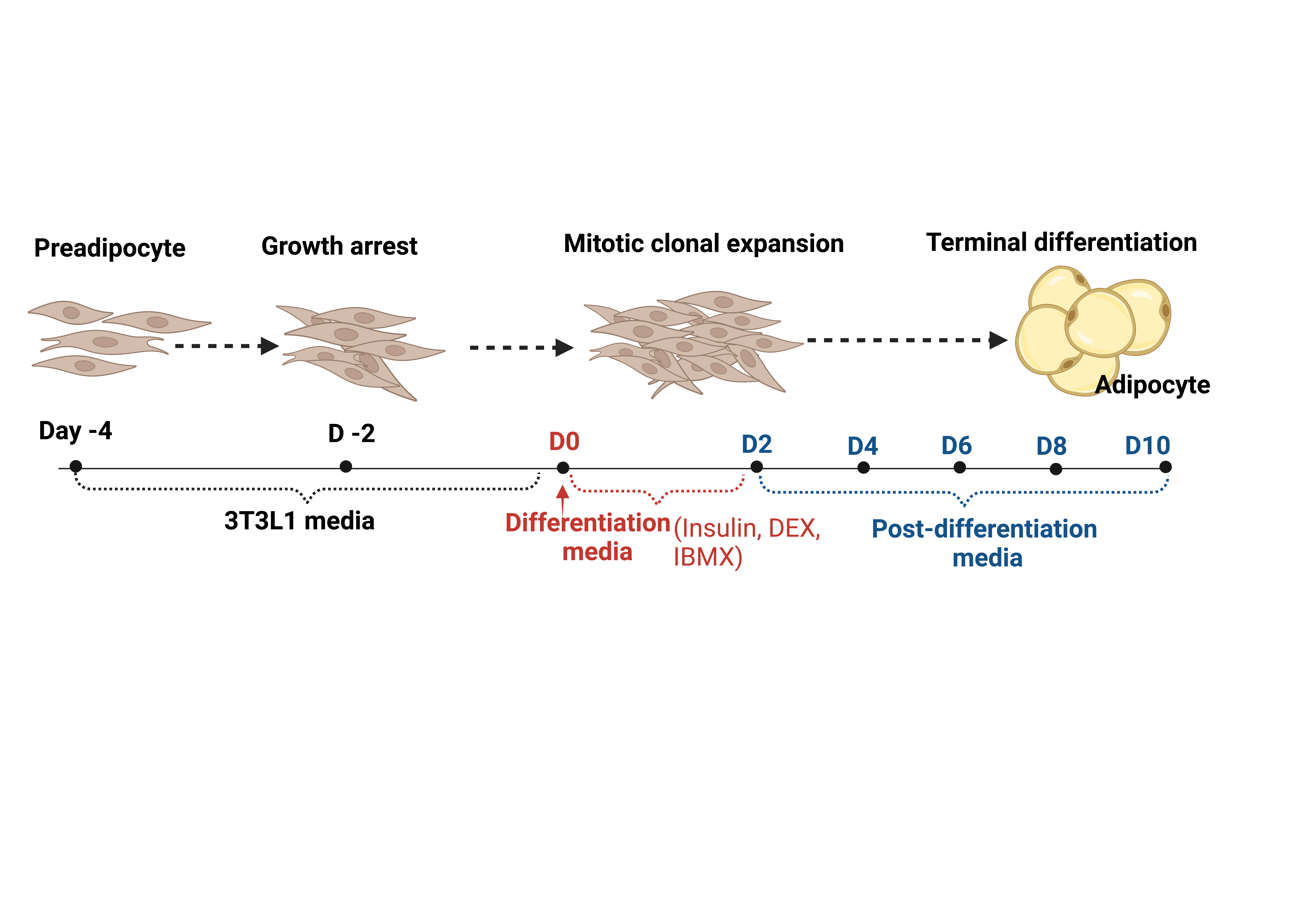


Supplementary Figure 9

Supplementary Figure 10

Supplementary table 1: Primer sequence details.

| Gene | Primer sequence | |
| --- | --- | --- |
|  | Forward (5’-3’) | Reverse (5’-3’) |
| CEBP-α | TTCATGGAGAATGGGGGCAC | GGCTGGCGACATACAGTACA |
| CEBP-β | GCTGAGCGACGAGTACAAGA | CTTGAACAAGTTCCGCAGGG |
| PPAR-γ | ATTGAGTGCCGAGTCTGTGG | GCCCAAACCTGATGGCATTG |
| COL1A1 | ACGCCATCAAGGTCTACTGC | ACTCGAACGGGAATCCATCG |
| COL6A3 | ACTGGAACCACGGAAGTTCA | GTCACTTCCAACATCGAGGC |
| DKK3 | GACCAGCAGATGCTATGCAC | TCACAGATGGTCCCATTGCC |
| GLUT4 | GCCTGCCCGAAAGAGTCTAAA | TGATGCCTGAGAGCTGTTGG |
| GPNMB | TCTATCCCTGGCAAAGACCCA | GCCCTTTCAAGGTTTGTACAGC |
| NLRP4 | GCCACATACGTACTTGGATTGC | TGCGCCAGAATTTCCATCCT |
| Pet2 | TCTCTCATTTTTAAGTGGGACAAC | TGCAGCATTTGTGTAGCTCT |
| Pla2G5 | GAAGAAGAGGAAATGAGGTGCTTG | TTTGTAGCCTGGTCTCAGCG |
| TSPAN10 | GGACTGGCAACAGAACCTGTACTT | GGTGTTCACTACAGCTCCGTCT |
| UHRF1 | TAGTGCTCACTTGGGTCTTCAG | CACATGATGCCGATGTACTCTCT |
| MMP13 | TGTTTGCAGAGCACTACTTGAA | CAGTCACCTCTAAGCCAAAGAAA |
| MMP3 | ACATGGAGACTTTGTCCCTTTTG | TTGGCTGAGTGGTAGAGTCCC |
| ADAM10 | CATTACACCAAAAACACCAGCG | TCGTAGGTTGAACTGTCTTCCA |
